# Unveiling the Antifouling Potential of Stabilized Poly(phosphorus ylides)

**DOI:** 10.1021/acsmacrolett.3c00524

**Published:** 2023-11-13

**Authors:** Dimitrios Karagrigoriou, Bela B. Berking, Qi Wang, Dulce M. Sánchez-Cerrillo, Daria R. Galimberti, Daniela A. Wilson, Kevin Neumann

**Affiliations:** Institute for Molecules and Materials, Radboud University, Heyendaalseweg 135, 6525 AJ Nijmegen, The Netherlands

## Abstract

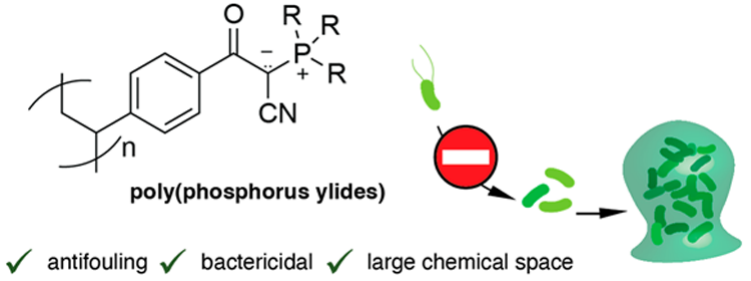

Zwitterionic polymers have emerged as highly attractive building blocks for antifouling coatings in biomedical applications. Notably, these polymers offer effective alternatives to the widely used poly(ethylene glycol) (PEG), which has raised concerns regarding its immunotoxicity and the development of PEG-specific antibodies. Polymeric ylides, a largely overlooked class of zwitterionic polymers, have been reported as effective antifouling scaffolds. However, the reported subclasses, poly(sulfur ylides) and *N*-oxides, lack structural diversity and chemical variability. In this study, we present the synthesis and characterization of polymeric phosphorus ylides as an unexplored class of poly(ylides) with significantly increased structural diversity, which is of high value when designing future ylide-based antifouling materials. Our findings demonstrate that, owing to their low dipole moments and hydration layers, these polymeric phosphorus ylides significantly reduce bacterial attachment. Furthermore, we observe selective toxicity toward bacteria rather than mammalian cells. The bactericidal nature of poly(phosphorus ylides), coupled with their expanded chemical space, provides a distinct advantage over existing materials, including zwitterionic polymers from betaine scaffolds. We anticipate that these unexplored structures will broaden the scope of antifouling applications for poly(ylides).

Formation of biofilms through nonspecific adsorption of biomolecules and microorganisms on surfaces possesses a significant threat to various medical applications.^1,2^ Because of their high resistance, combating infections related to biofilms requires more aggressive antibiotic treatments.^3,4^ Once attached, bacteria irreversibly adhere to the surfaces, creating a pool of cells that continuously recruit more cells and thereby actively contribute to the infection process.^5^ Importantly, the nonspecific adhesion of organic and biological materials also affects the efficacy of nanomedicinal devices.^6^ For instance, when serum proteins adsorb onto the surfaces of drug nanocarriers, they trigger an immune response, leading to rapid clearance and reducing the circulation half-life of the drug delivery devices.^7,8^

Polyethylene glycol (PEG) has been commonly used to prevent the adhesion of biomolecules and bacteria by inducing hydration layers.^9,10^ These hydration layers act as a physical barrier, making adhesion unfavorable because of the replacement of water. Recent reports have highlighted the complex behavior of PEG, including the insertion of chemical anchors, which is undesirable when developing antifouling coatings.^11,12^ Concerns regarding immunotoxicity and the development of PEG-specific antibodies have led to the emergence of materials from zwitterionic polymers as alternatives to PEG.^13−15^ These materials, which typically derive from hydrophilic polybetaines, mimic the naturally occurring phosphorylcholine and have extensive applications as antifouling coatings.^16^ Unlike PEG, which forms hydration layers through hydrogen bonds, zwitterionic polymers are believed to form hydration layers based on stronger electrostatic interactions.^17^ We would like to emphasize that although polymeric zwitterions have been reported to reduce immune responses, there are recent reports suggesting that certain zwitterionic polymers can display similar levels of immunotoxicity as PEG.^18−20^

Recently, our group and the group of S. Jiang have independently reported poly(ylides) as a new class of unexplored zwitterionic materials (Figure 1).^21−23^ This long-overlooked class of polymers features some distinct structural properties, namely, a positive charge directly adjacent to a negative charge, thereby being in strong contrast to polybetaines that typically have a carbon spacer between the charges. The Jiang group disclosed *N*-oxides and demonstrated their ultralow antifouling properties in mice models.^22^

**Figure 1:**
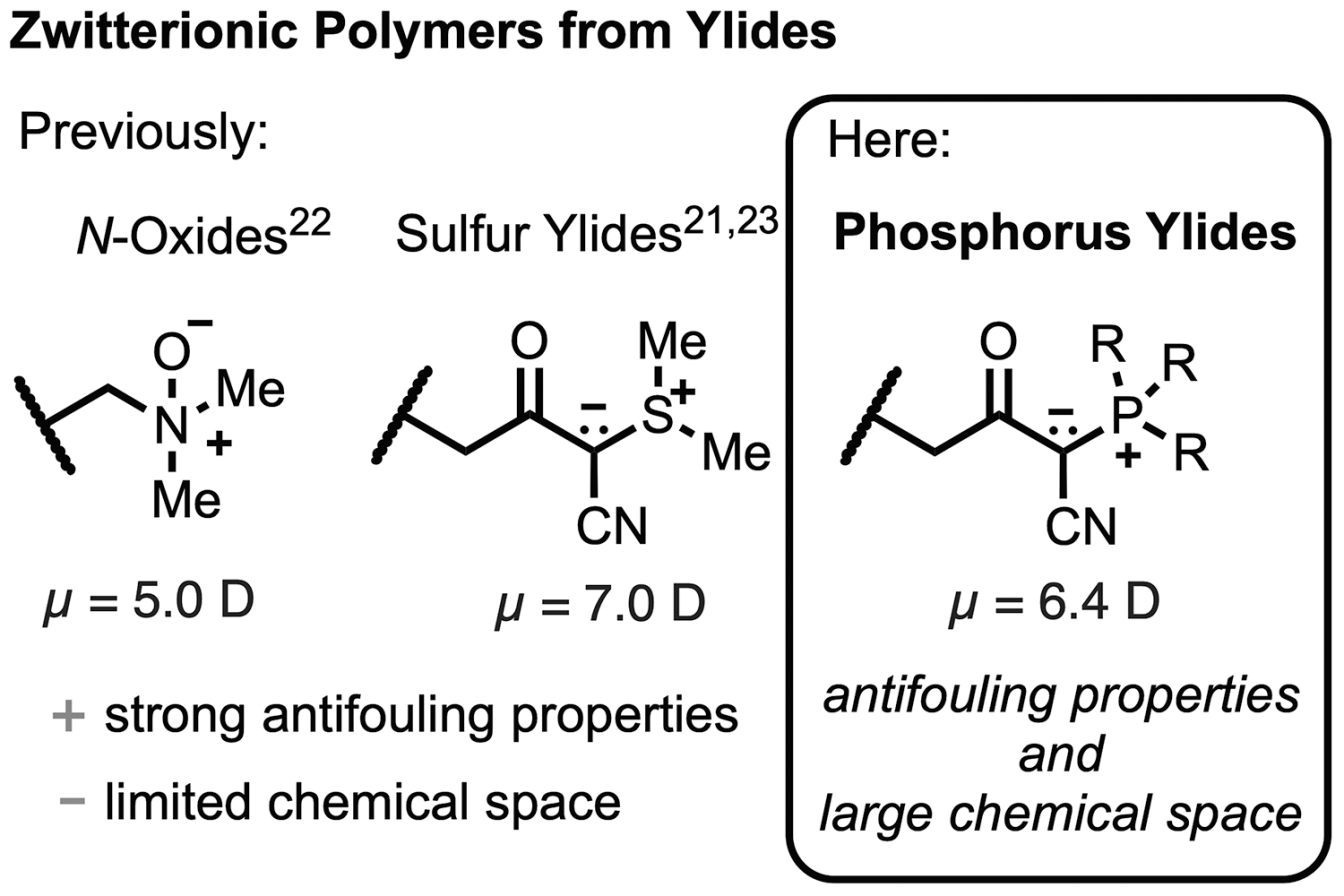
Previously reported zwitterionic polymers from ylide building blocks have shown excellent antifouling properties but are severely limited in their chemical space. Among other reasons, a reduced dipole moment is believed to be crucial for showing strong antifouling behavior. Here, we report poly(phosphorus ylides) that offer a wide and unexplored chemical space while maintaining excellent antifouling properties (R = Me, Ph).

Yet, the restricted structural diversity and chemical space of *N*-oxides hamper further advancements. Our group explored the use of sulfur ylides as the zwitterionic residue for antifouling materials, where the negative charge is localized on a carbon and the positive charge on a sulfonium residue.^21,23^ Our results indicated that coatings from poly(sulfur ylides) not only induce a strong hydration layer but also provide a hostile environment selectively to bacteria. Consequently, we observed that those bacteria that overcome the physical barrier of hydration experience a bactericidal environment.

Here, we sought to provide stabilized phosphorus-based ylides as a new form of polymeric ylides that would significantly expand the chemical space available for designing new ylide-derived materials for medical applications (Figure 1). In addition, the investigation of new zwitterionic scaffolds would provide deeper understanding of structure–properties relationships, essential for molecular tailoring of antifouling materials in the future. In a recent study, T. Wei et al. investigated the effect of linker lengths on the ability to prevent biofilm formation and revealed that—besides a strong hydration layer—a reduced dipole helps to prevent dipole/dipole interactions with biomolecules, ultimately leading to a reduced adhesion of microorganisms.^24^ The authors identified that the dipole of zwitterionic *N*-oxide residues is significantly lower than the dipoles of betaines, including the frequently used carboxy-*N,N*-dimethylammonium residues. While the dipole moment alone is not sufficient to explain antifouling properties, it rationalizes the strong antifouling properties observed for ylide-containing polymers. This finding encouraged us to determine the dipole of our recently reported zwitterionic poly(sulfur ylides) for comparison with the reported *N*-oxides. Indeed, the zwitterionic sulfur ylide headgroup displayed a dipole (7.0 D) similar to that of the reported *N*-oxides (5.0). While slightly stronger, the dipole is still significantly lower in comparison to the conventionally employed betaine residues (e.g., carboxy-*N,N*-dimethylammonium with D = 13.9 D).^24,25^ Interestingly, acyl-stabilized phosphorus ylides exhibit a lower dipole moment (<7.0 D). For that reason, we hypothesized that polymeric phosphorus ylides would be a powerful addition to the toolbox of polymeric ylides offering similar antifouling properties as the previously reported *N*-oxides and sulfur ylides while significantly enhancing the available chemical space. Inspired by the early work of H. Wasserman, we accessed two different types of stabilized phosphorus ylide building blocks, namely styrene-derived triphenyl phosphorus ylide **1** and trimethyl phosphorus ylide **2** from triphenyl phosphine and trimethyl phosphine, respectively.^26,27^ When polymerizing triphenyl phosphorus ylide **1** using a dithioester as the RAFT agent (2-[[(2-carboxyethyl)sulfanylthiocarbonyl]-sulfanyl]propanoic acid), we observed a prolonged delay-time in which no significant consumption of monomer was detected. Notably, after 120 min, controlled polymerization was observed, confirmed by the narrow PDI of P(TPPY) **4** (Figure 2). One explanation of this delay-time prior to the polymerization could be the large steric bulk provided by the triphenyl phosphonium residue hindering rapid polymerization. In this case, only upon seed formation of supramolecular assemblies an efficient polymerization is induced. In strong contrast, trimethyl phosphorus ylide **2** could be rapidly polymerized employing the same RAFT agent as determined by ^1^H NMR and GPC. By monitoring the polymerization with ^31^P NMR, we observed the formation of trace amounts of trimethyl oxide during the polymerization. Indeed, when polymerizing monomer **2** at 70 °C, less oxidation was observed, yet the rate of polymerization decreased significantly, which in turn resulted in similar amounts of oxidized byproduct over time. While we determined the total amount of affected monomers in the polymer to be <2%, we believe that future studies are necessary to determine the exact nature and mechanism of this side-reaction. Notably, the resulting homopolymers poly(trimethyl phosphorus ylides) (P(TMPY)) **6** and **7** showed great solubility in polar organic solvents, which is in strong contrast to polystyrene or the more hydrophobic poly(triphenyl phosphorus ylides) copolymer and homopolymer PS-*co*-P(TPPY) **3** and P(TPPY) **4**. Stability is a crucial prerequisite for the development of sufficient antifouling properties. In order to determine the stability of the phosphorus ylide residue in relevant biological media, we incubated **2** in BHI media at 37 °C over 24 h. Analysis with ^31^P NMR revealed that the phosphorus ylide residue is stable under such conditions (ESI 2.6).

**Figure 2:**
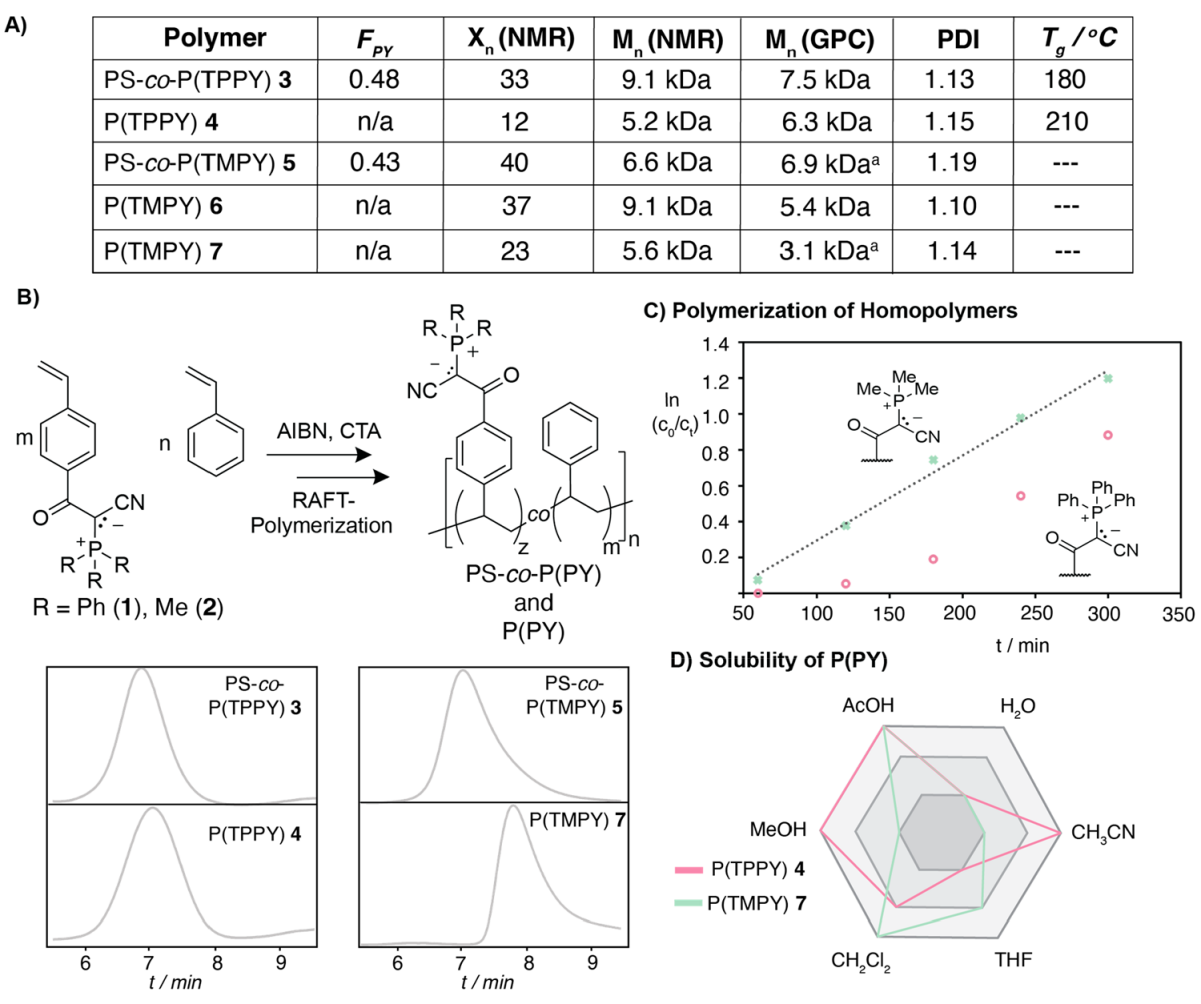
A) Polymers accessed from phosphorus ylide building blocks **1** and **2**. B) Phosphorus ylide building blocks **1** and **2** are polymerized using RAFT agent (2-[[(2-carboxyethyl)sulfanylthiocarbonyl]-sulfanyl]propanoic acid) and AIBN w/o styrene as a co-monomer. Analysis with GPC shows that all polymers can be obtained with a mono-modal distribution and narrow PDI. C) Polymerization was monitored via ^1^H-NMR and ^31^P-NMR. Reaction monitoring revealed a delay-phase when monomer **1** was used alone, while monomer **2** can be polymerized in a controlled manner. D) Solubility of homo-polymers **4** and **7** differ greatly from each other. While P(TPPY) **4** shows similar behavior to polystyrene, P(TMPY) **7** is soluble in more polar solvents. GPC is measured in dimethyl acetamide.

To further investigate the ability of P(TMPY) to induce hydrophilicity to surfaces in a similar manner as it was shown for the recently reported poly(ylides) and poly(betaines), glass slides were coated with polymers **3**, **4**, **5**, and **7**, and water contact angles were determined. As expected, both hydrophobic polymers bearing triphenyl phosphonium moieties **3** and **4** displayed contact angles of 87° and 81°, respectively, indicating a rather hydrophobic surface. In contrast, surfaces coated with trimethyl phosphonium bearing polymers **5** and **7** exhibited more hydrophilicity and contact angles of 73° and 50°, respectively (Figure 3). Surface energy analysis revealed a strong Lewis base component γ_s_^–^ for P(TMPY) **7**, namely 26.7 mJ m^–2^, which indicates relatively tightly bond water molecules.^28^ This aligns with our previous observation that acyl/nitrile-stabilized ylides display strong hydrogen bond acceptor capabilities.^21^ In addition, the surface energy γ_s_ was determined as 49.8 mJ m^–2^, which indicates a rather hydrophilic surface environment.^29^ Hydrophilic surfaces have the possibility to repeal biomolecules by forming hydration layers acting as a physical barrier.^30,31^ However, T. Wei et al. have impressively shown that antifouling properties cannot be explained with hydration alone, but other parameters need to be taken into account such as dipoles and toxicity.^24^ With these considerations in mind, we investigated the ability of poly(phosphorus ylides) to prevent the formation of bacterial biofilms. For this purpose, we covalently immobilized P(TMPY) **7** on otherwise hydrophobic polystyrene surfaces using EDC/NHS coupling chemistries. In a similar manner, we attached zwitterionic P(DMAPS) **8** on surfaces. This is because P(DMAPS) is often employed as a state-of-the-art zwitterionic polymer for antifouling coatings.^32,33^ First, we evaluated these coatings in their ability to prevent the accumulation of biomass upon incubation with *Pseudomonas aeruginosa*.

**Figure 3:**
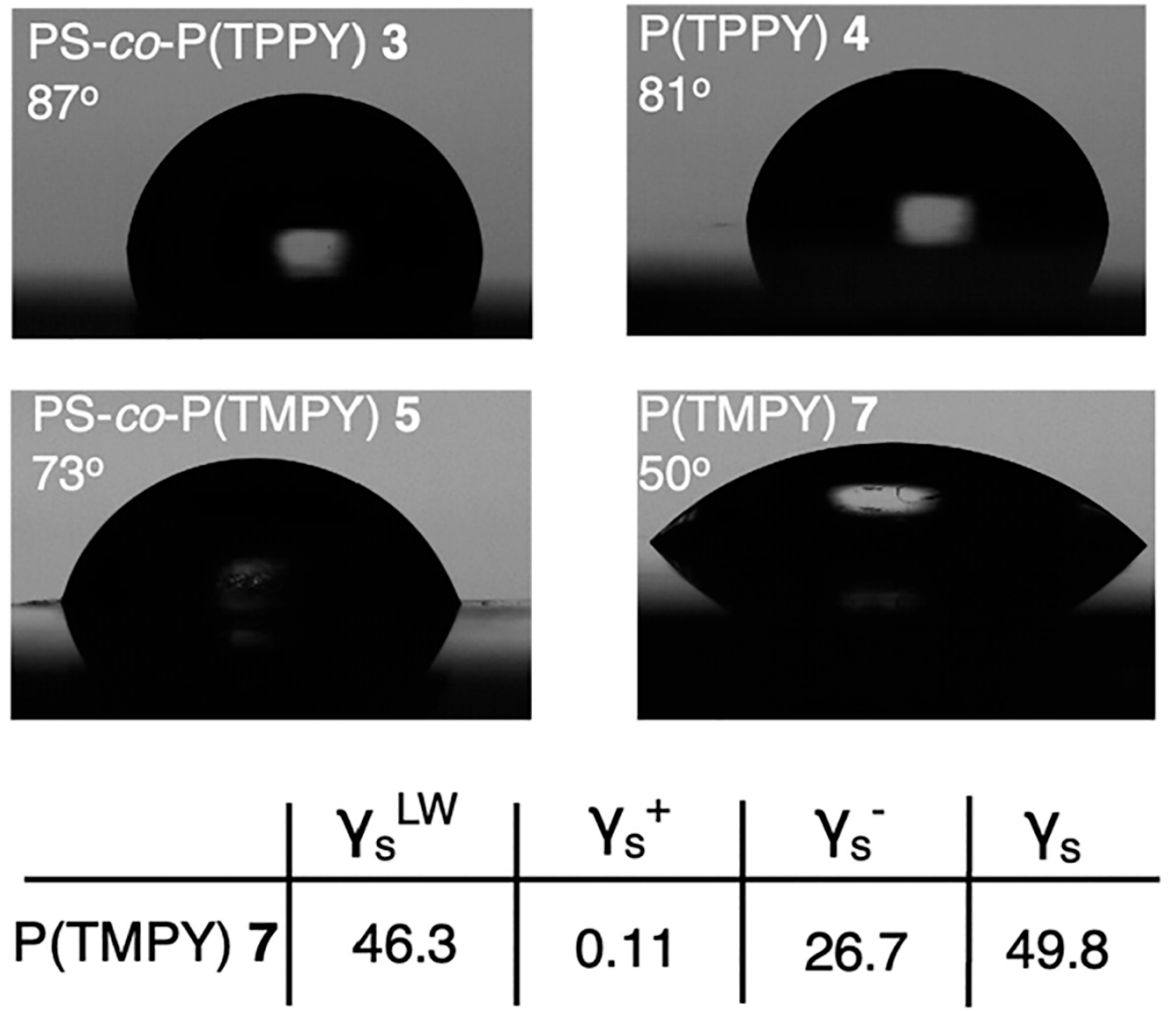
Water-contact angles of polymers **3**, **4**, **5**, and **7** were measured on glass slides. Surface energy analysis of P(TMPY) **7** reveals a high Lewis acid component, indicating tightly bound water molecules.

Both coatings displayed a significant decrease in biomass in comparison to simple polystyrene surfaces, while the difference between P(TMPY) **7** and P(DMAPS) **8** was statistically not significant. In contrast, when determining the ratio of viable and dead cells, a significant difference was observed. Surfaces coated with poly(betaine) **8** and untreated polystyrene displayed a majority of viable cells without statistical significance; yet, poly(phosphorus ylide)-coated surfaces exhibited a majority of dead cells. These results indicate that poly(phosphorus ylides) behave in a similar way than poly(sulfur ylides) by preventing biofilm formation with a combination of hydration layer and toxicity toward bacterial cells. For many applications, a selective toxicity toward bacterial cells is desirable, for example, when working with implants or other biomedical devices. For that reason, we determined the effect of polymeric phosphorus ylides on mammalian cell lines. Three cell lines, HEK293T, NIH3T3, and CHO, representing human embryonic kidney, mouse fibroblasts, and epithelial ovarian cell lines, respectively, were incubated with P(TMPY) **7** and small molecule analogue **9** for 72 h (Figure 4 and ESI). At lower concentrations, no cytotoxicity was observed for either of the phosphorus ylide species. Whereas small molecule phosphorus ylide **9** became cytotoxic for fibroblast cells at a higher concentration of 1 mg/mL, P(TMPY) **7** only displayed reduced viability (60% viability). The difference in toxicity between polymeric ylide **7** and small molecule **9** could be related to enhanced cellular uptake of the small molecule. Notably, the ylides do not seem to affect CHO cells.

**Figure 4:**
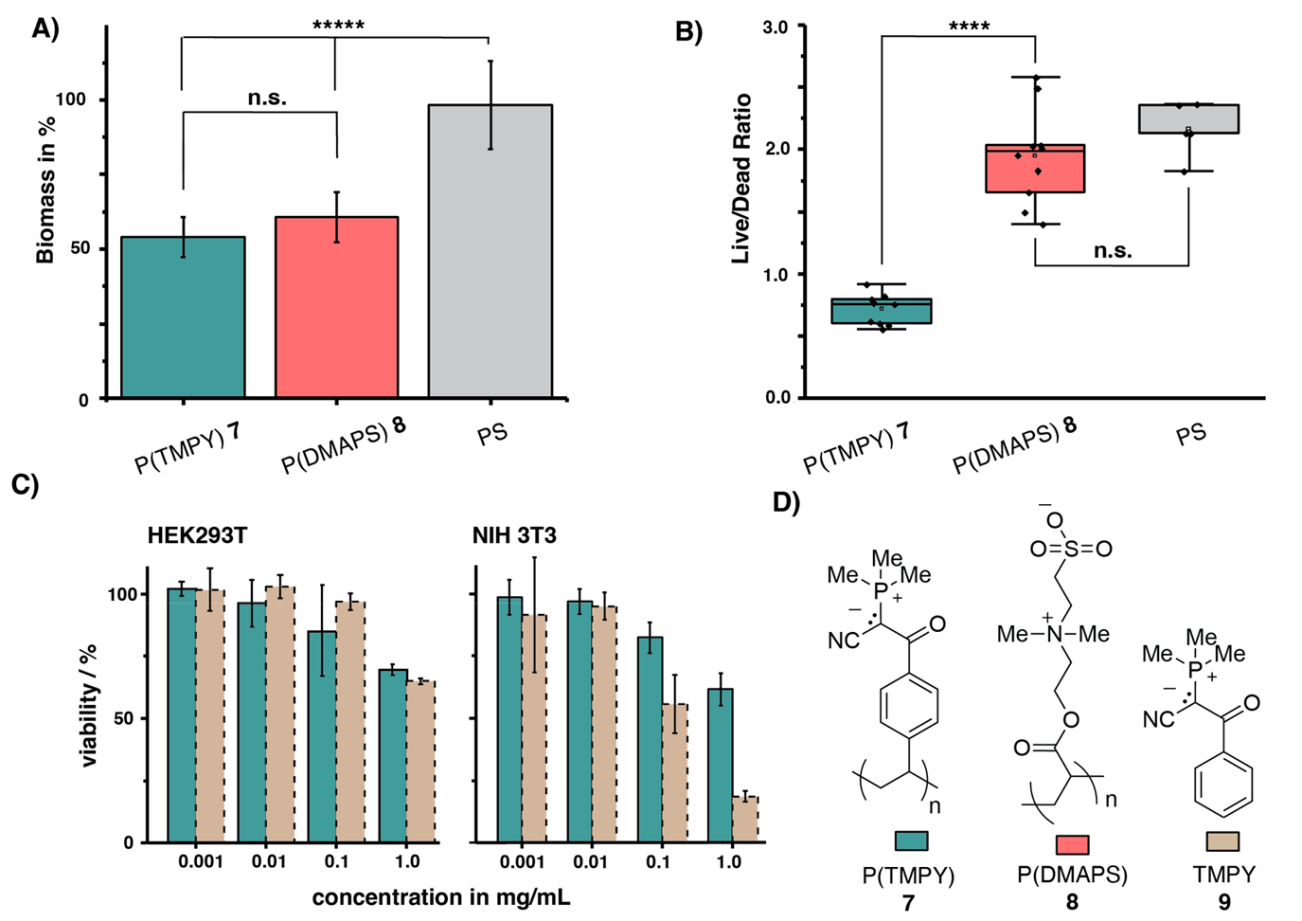
A) Biomass formation upon incubation with *Pseudomonas aeruginosa* (4 h, 37 °C) on surfaces covalently coated with zwitterionic P(TMPY) **7** and P(DMAPS) **8** alongside the reference of polystyrene. B) Live/dead ratio was determined by applying Syto9 and PI stain upon incubation of samples with *Pseudomonas aeruginosa* (4 h, 37 °C). C) Cytotoxicity was determined by incubating HEK293T and NIH 3T3 cells with polymeric phosphorus ylide **7** or small molecule analogue **9** for 72 h at 37 °C. D) Structures of P(TMPY) **7**, P(DMAPS) **8**, and small molecule TMPY **9**.

We hypothesize that the principle of ‘amphiphilic balance’ may be applicable to the here reported poly(phosphorus ylides) and explains the preferential toxicity to bacterial cells over mammalian cells.^34,35^ While ylide residues possess hydrophilic characteristics, the polystyrene backbone exhibits significant hydrophobicity. This structural attribute facilitates an initial interaction with the bacterial membrane through electrostatic attraction, with subsequent insertion of the hydrophobic component into the cell membrane, which ultimately leads to membrane permeabilization.^36^

Taken together, we have introduced poly(phosphorus ylides) as a readily available and versatile class of zwitterionic polymers bearing acyl/nitrile-stabilized carbon ylide-residues. Surface energy analysis indicates that poly(trimethyl phosphorus ylides) induce a surface hydration layer. In addition, bacterial assays show that surfaces coated with poly(trimethyl phosphorus ylides) exhibit selective toxicity toward bacterial cells. In contrast to existing betaine-derived zwitterionic polymers, polymeric ylides have a positive charge directly adjacent to the negative charge, thereby offering a significantly decreased dipole moment. In addition, we have shown that polymeric phosphorus ylides provide a wide unexplored chemical space. By offering a second mechanism that goes beyond a simple repellent barrier, phosphorus ylides are set apart from the conventionally employed zwitterionic polymers such as poly(DMAPS). We believe that the readily available and chemically versatile class of poly(phosphorus ylides) will find many new applications in the field of medicine, material chemistry and nanotechnology.

## Supporting Information Available

Supporting figures and synthetic schemes, NMR spectra, GPC data, and DSC traces (PDF)
