# Supplementary material for: Unveiling the Antifouling Potential of Stabilized Poly(phosphorus ylides)
Source: ACS Macro Lett. 2023 Nov 13;12(12):1608–13. doi: 10.1021/acsmacrolett.3c00524 (PMC10734299; doi:10.1021/acsmacrolett.3c00524)
Supplement: Supplementary file 1 — mz3c00524_si_001.pdf [file mz3c00524_si_001.pdf]

Supporting Information

Unveiling the Antifouling Potential of Stabilized Poly(phosphorus ylides)

D. Karagrigoriou, B. Berking, Q. Wang, D. M. Sánchez-Cerrillo, D. Galimberti,

D. A. Wilson*, K. Neumann*

Institute for Molecules and Materials,

Radboud University, Heyendaalseweg 135, 6525 AJ Nijmegen, The Netherlands

kevin.neumann@ru.nl

1. Materials

Reagents were obtained from Sigma Aldrich/Merck (Zwijndrecht, The Netherlands), Fluorochem BV (Amsterdam, The Netherlands) and TCI Europe (Zwijndrecht, Belgium) and were used without purification unless otherwise stated. Vinyl benzoic acid was obtained from Carbosynth. Solvents were obtained from VWR, Fisher, Acros Organic and Sigma Aldrich/Merck. Solvents were dried by passing over activated alumina columns in a MBraun MB SPS800 under a nitrogen atmosphere and stored under argon. Reactions were carried under air unless stated otherwise. Typically, such air-sensitive reactions were carried out under atmosphere of nitrogen using Schlenk technique. Ultrapure Milli-Q water was obtained from QPOD Milli-Q system. Reactions and fractions from flash column chromatography were monitored by thin layer chromatography using glass TLC plates (Merck, TLC Silica gel 60 F_{254}) and if necessary visualized by staining with $KMnO_4$ solution. Column chromatography was performed on VWR SiO_2 Type (40-63 mesh) using a forced flow of air at 0.5-1.0 bar.

2. Instrumentation

Nuclear Magnetic resonance (NMR) characterization was carried out on a Bruker AVANCE HD nanobay console with a 9.4 T Ascend magnet (400 MHz) and a Bruker AVANCE III console with a 11.7 T UltraShield Plus magnet (500 MHz) equipped with a Bruker Prodigy cryoprobe, in chloroform ($CDCl_3$) or DMSO-d_6. NMR spectra were recorded at 298 K unless otherwise specified. Chemical shifts are given in parts per million (ppm) with respect to tetramethylsilane (TMS, δ 0.00 ppm) as internal standard for ^{1}H NMR. Coupling constants are reported as J values in Hz. Peak assignment is based on 2D COSY, ^{1}H−^{13}C HSQC, and ^{1}H−^{13}C HMBC spectra. The splitting patterns are indicated as follows: s, singlet; br. s, broad singlet; d, doublet; t, triplet; m, multiplet. Gel permeation chromatography (GPC) equipped with PL gel 5 μm mixed D column calibrated for polystyrene (580− 377400 g/mol) was carried out on a Shimadzu instrument with NMP or DMA as eluent using differential refractive index and UV absorbance (254 nm).

2. Supporting Figures

2.1 Dipole calculations

The static dipole moments of model compounds displaying N-oxide, sulfur ylide and phosphorus ylide have been computed with the Gaussian16 code.[1] The B3LYP[2,3] functional, augmented with the Grimme D3 dispersion term,[4] and the 6-311++G** basis set have been chosen. The geometry has been fully optimized before computing the dipole (Figure 1).

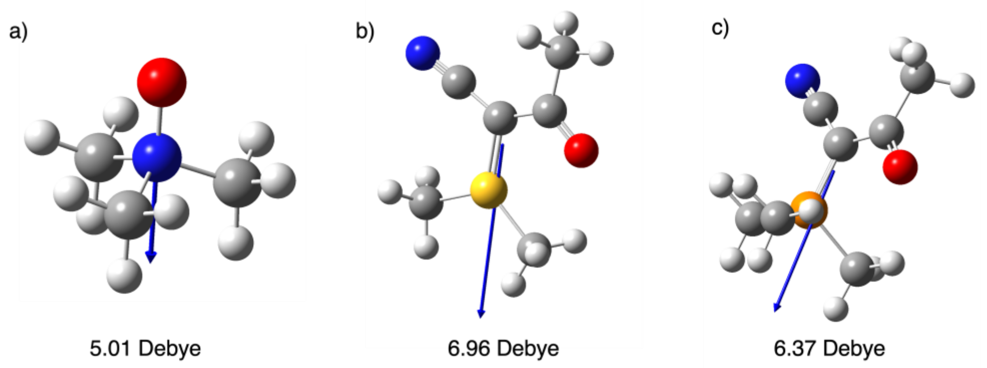

Figure S1. Structure and dipole of **a)** N-Oxide, **b)** sulfur ylide and **c)** phosphorus ylide.

2.2 Synthetic schemes

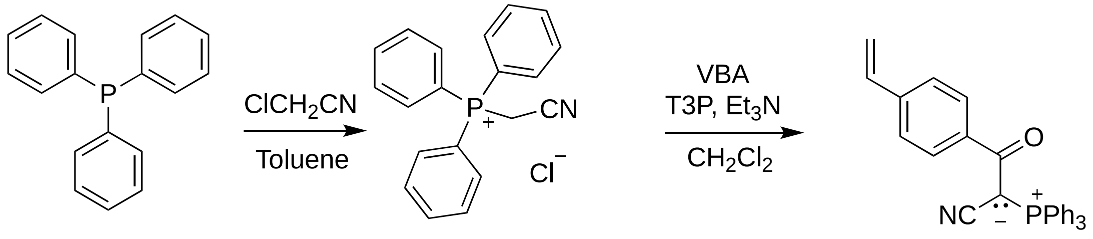

S1

1

Figure S2. Synthetic route to access triphenyl phosphorus ylide **1** from triphenyl phosphine.

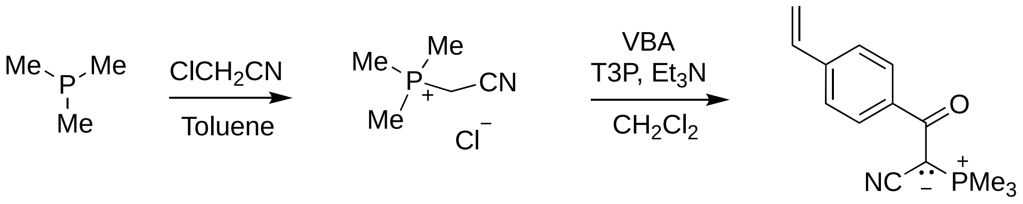

S2

2

Figure S3. Synthetic route to access triphenyl phosphorus ylide **2** from triphenyl phosphine.

2.3 Stability

In order to assess the stability of phosphorus ylide, small molecule PY **S3** was incubated in varying conditions and the stability was investigated using ^{31}P-NMR after 24 h. PY **S3** was incubated at room temperature and left on the bench exposed to daylight. Only upon treatment with 4M NaOH, degradation was observed, namely formation of triphenyl oxide.

Table S1. Overview of stability assays performed using small molecule PY **S3**.

**Condition**	**Stability**
Acetic Acid	> 98 %
Piperidine	> 98 %
Acetic acid anhydride	> 98 %
Aq. NaOH (4M)	< 2 %
PBS pH 7.4 (100 mM)	> 98 %
Borate buffer pH 8.8 (50 mM)	> 98 %

2.4 Solubility

Solubility of polymeric phosphorus ylides was tested in various solvents at room temperature by dissolving 1 mg of the polymer in 1 mL of solvent. The mixtures were vortexed for a while and the solubility was determined by visual appearance. If the polymer did not dissolve, then it was sonicated for 10 min with heating and then the solubility was again checked.

Table S2. Solubility of polymeric phosphorus ylides in different solvents, green = soluble (> 1 mg/mL), yellow = partly soluble (1 mg/mL > solubility > 0 mg/mL), red = insoluble (no solubility observed).

**Polymer**	Water	THF	Et_2O	DMSO	DCM	Toluene	ACN	MeOH	Acetic acid
P(TPPY) **3**	red	red	red	green	green	yellow	red	red	green
PS-*co*-P(TPPY) **4**	red	yellow	red	green	green	yellow	red	red	green
P(TMPY) **7**	red	red	red	green	yellow	red	green	green	green
PS-*co*-P(TMPY) **5**	red	yellow	red	green	green	yellow	green	yellow	green

2.5 DSC

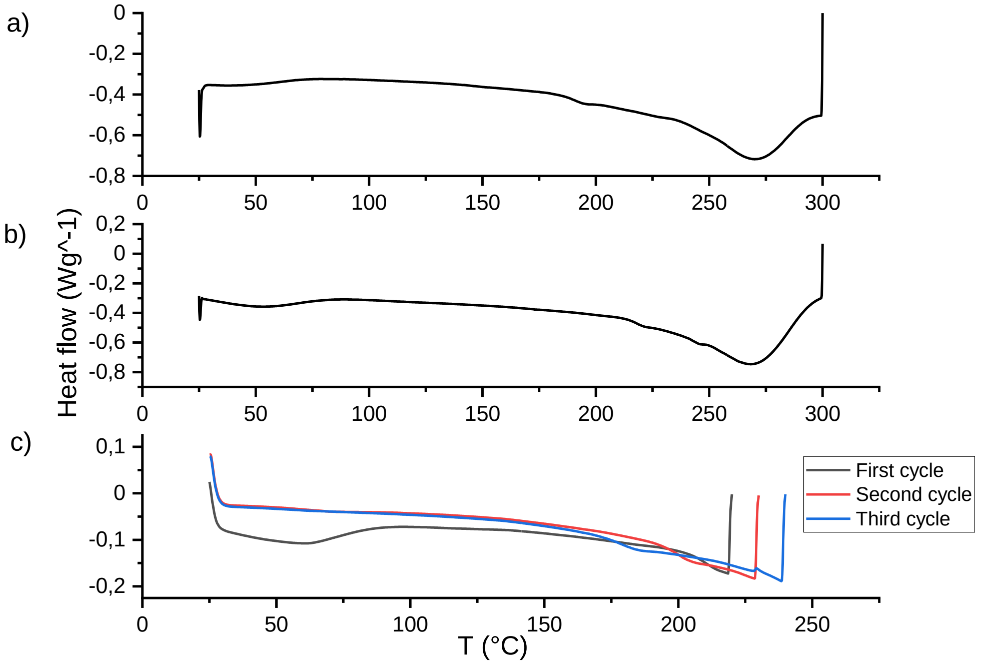

Figure S4. DSC traces of **a)** PS-co-(TPPY) **3**, **b)** P (TPPY) **4** and c) P(TPPY) **4**. For a) and b), the second cycle is displayed; whereas for **c)** all three cycles are displayed of P(TPPY) **4** for comparison.

2.6 Surface-Attachment of polymeric ylides

For surface attachment, we used amine-coated well plates that were obtained from biomat (MCB02F-AM1). Well-plates were modified with polymers that bear carboxylic acids using the following protocol. In brief, carboxylic acid containing polymers were dissolved in THF/H_2O (1:3, v/v, 10 mM) and EDC (3 equiv) with NHS (5 equiv) were added. The solution was stirred for 10 min and added to the well plates. After 4 hours, the solution was removed and the wells were thoroughly washed with water, EtOH, water/THF, water and finally with EtOH again. Finally, the well-plates were air-dried overnight. Because of the autofluorescence of polymeric phosphorous ylides, it was possible to confirm the modification by measuring fluorescence (λ_{ex} = 380 nm, λ_{em} = 450 nm, bandwidth 20 nm).

2.7 Stability of Phosphorous Ylide residue in BHI Media

For determining stability of phosphorous ylide residue, compound 2 was incubated in BHI media at 37°C for 24 hours. Stability was determined with ^{31}P-NMR at different time points (Figure S5).

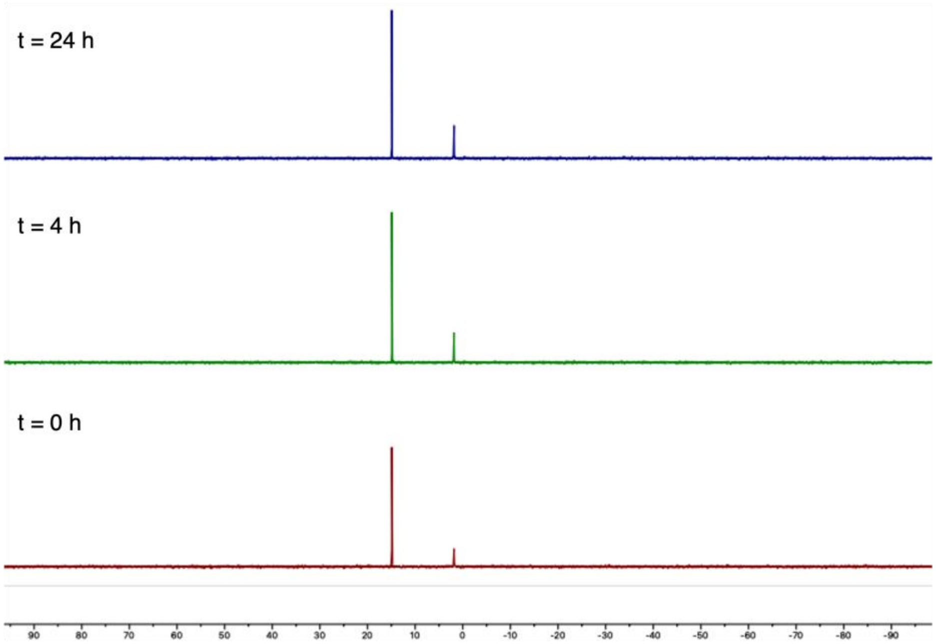

Figure S5. 31P-NMR spectra of incubation of 2 in BHI media at 37°C. Signal at 0 ppm corresponds to residual PO_4^{3-} from BHI buffer.

2.8 Bioassays

2.8.1 Crystal Violet Stain

96 well plates with various modified surfaces were inoculated with 100 µl of bacterial solution (strain ATCC 10145) in BHI broth (OD 0.005) and incubated for 4 hours at 37 °C to allow for adhesion and biofilm formation. After 4 hours, all wells were gently washed three times with 1x PBS buffer (pH 7.4) to remove planktonic cells and subsequently stained for 10 minutes with 0.01 % Crystal violet in water (w/v). CV solution was then removed, and wells washed three times with PBS buffer before letting the well plate dry overnight for analysis. Stained biomass was resolubilized in 30% Acetic acid in distilled water (v/v) and transferred to a new clear bottom well plate. Absorbance was measured at 590 nm in a Tecan Spark M10 plate reader.

2.8.2 Live/Dead Assay

96 well plates with various modified surfaces were inoculated with 100 µl of bacterial solution in BHI broth (OD 0.005) and incubated for 4 hours at 37 °C to allow for adhesion and biofilm formation. After 4 hours, all wells were gently washed three times with 1x PBS buffer (pH 7.4) to remove planktonic cells. BacLight stain (Molecular Probles) containing Syto9 and Propidium Iodide was used to create a suitable working solution: for Syto9 a final concentration of c = 11.1 nM and for Propidium Iodide a final concentration of c = 66.6 nM in PBS (150 mM NaCl, 100 mM $NaPO_4$ mM, pH 7.4). Wells were stained for 10 minutes and washed three times with PBS (150 mM NaCl, 100 mM $NaHPO_4$, pH 7.4). Fluorescence intensity was measured at λ_{ex} = 485 nm, λ_{em} = 535 nm and λ_{ex} = 300 nm, λ_{em} = 632 nm, respectively, with a bandwidth of 20 nm, 30 flashes and an Integration time of 40 µs using a Tecan Spark M10 plate reader.

2.8.3 Cytotoxicity assays

HEK293T, Chinese Hamster Ovarian (CHO) and NIH 3T3 cells were cultured in DMEM medium supplemented with 10% FBS. After cells reached a confluence of around 50 %, they were rinsed with 1x PBS three times and detached with 4 ml Trypsin for 3 minutes. Trypsin was quenched by adding 8 ml of DMEM medium. The cells were transferred to a 15 ml falcon and spun down 5 minutes at 0.3 rcf. The supernatant was discarded, and cells seeded with DMEM complete medium in a 96 well plate at a density of 4.0 to 4.5 x 10^4 cells/ml and incubated for 24 hours at 37 °C with 5 % CO_2. Afterwards, varying concentrations of polymer in DMEM complete medium were added to the wells and left to further incubate at 37 °C and 5 % CO_2 for 72 hours. 10 µl of CCK8 (Sigma Aldrich) was added to the wells, incubated for 3 hours and the absorbance measured at 450 nm.

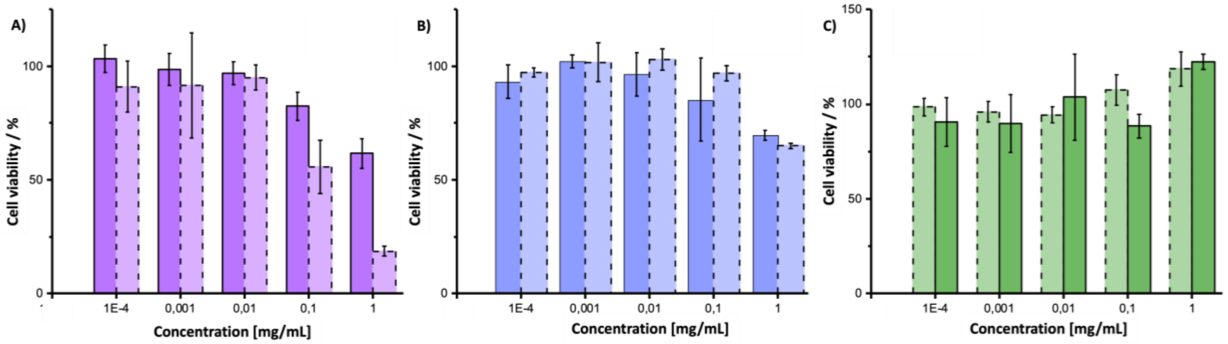

Figure S6. Cytotoxicity of mammalian cells after 72 h incubation with P(TMPY) **7** (solid line) and small molecule analogue **9** (patterned line) **A)** NIH 3T3 **B)** HEK 293 **C)** CHO.

2.8.4 Bacterial toxicity assays

P. aeruginosa cultures were diluted to an OD of 0.005 in BHI supplemented with Tyloxapol 0.04%. The bacterial solutions were incubated with varying concentrations of P(TMPY) **7** (1.0, 0.1, 0.01, 0.001 mg/mL) and added to clear wellplates. Absorbance (600 nm) was immediately measured to get a baseline, before returning the plate to the incubator for 24 hours at 37 °C. Finally, the absorbance was measured at 600 nm and data evaluated by subtracting the final OD from the baseline to retrieve an OD increase after 24 hours.

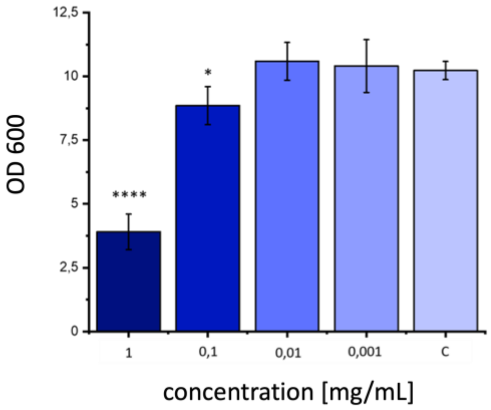

Figure S7. Growth factors of planktonic bacteria in the presence of 1 to 0.001 mg/ml over 24 hours. Samples were conducted in replicates of 10. P value ≤ 0.01.

3. Experimental protocols

3.1 Small molecules synthesis

Triphenyl phosphonium salt S1

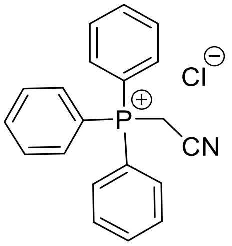

The triphenyl phosphonium salt **S1** was synthesized according to a literature protocol.[5,6] Triphenylphosphine (5.0 g, 19.1 mmol, 1.0 eq) was dissolved in dry toluene (30 mL) in a flame dried and Argon flushed Schlenk tube. Then chloacetonitrile (2.4 mL, 38.1 mmol, 2 eq) was added to the colorless solution and the reaction mixture was bubbled with Argon for 30min. The reaction mixture was stirred overnight at 60 ^{o}C under Argon atmosphere and a white precipitated was formed. The suspension was cooled to room temperature and the precipitate was collected by filtration under vacuum and washed with cold Et_2O. The product was obtained as a white solid (yield 57%). ^{1}H NMR (400 MHz, Dimethyl sulfoxide-d_6) δ 8.05 – 7.96 (m, 3H), 7.94 – 7.81 (m, 12H), 6.02 (d, J = 15.9 Hz, 2H).

Triphenyl phosphorus ylide monomer 1

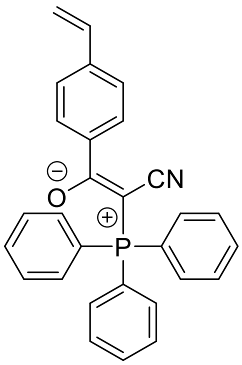

4-vinyl benzoic acid (1.15 g, 7.73 mmol, 1.0 eq) was dissolved in CH_2Cl_2 (47 mL) and the mixture was stirred. Triethylamine (3.3 mL, 23.2 mmol, 3.0 eq) was added followed by T3P (6.0 mL, 10.1 mmol, 1.3 eq). The mixture was stirred for 15 min and then the phosphonium salt **S1** (3.65 g, 10.8 mmol, 1.4 eq) was added. The reaction mixture was

stirred at room temperature overnight. It was then diluted with CH_2Cl_2 and it was washed with sat. $NaHCO_3$ (x1), H_2O (x1) and brine (x1). The crude product was dried over Na_2SO_4 and purified via column chromatography on silica gel eluting with CH_2Cl_2/MeOH mixtures (98:2 by volume) to obtain the product as a light brown solid (yield 20%). ^{1}H NMR (400 MHz, Chloroform-*d*) δ 8.04 – 7.96 (m, 2H), 7.75 – 7.68 (m, 4H), 7.68 – 7.66 (m, 2H), 7.66 – 7.57 (m, 3H), 7.57 – 7.47 (m, 6H), 7.47 – 7.39 (m, 2H), 6.73 (dd, J = 17.6, 10.9 Hz, 1H), 5.80 (d, J = 17.6, 0.9 Hz, 1H), 5.29 (d, J = 10.9, 0.9 Hz, 1H). ^{13}C NMR (101 MHz, Chloroform-*d*) δ 189.50, 189.46, 140.02, 138.36, 138.28, 136.45, 133.73, 133.63, 133.07, 133.04, 129.25, 129.12, 128.46, 125.85, 123.94, 123.01, 122.72, 122.57, 115.26, 49.32, 48.06. ^{31}P NMR (162 MHz, Chloroform-*d*) δ 22.16. HRMS (ESI): calculated for $[M+H]^+$: m/z 432.15173, found: m/z 432.15092.

Trimethyl phosphonium salt S2

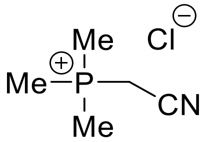

The trimethyl phosphonium salt was synthesized according to a literature protocol.[7] Trimethyl phosphine solution (15.0 mL, 15.0 mmol, 1.0 eq) was added to a flame dried and Ar flashed Schlenk tube. Chloroacetonitrile (1.42 mL, 22.5 mmol, 1.5 eq) was added to the solution of PMe_3 at 0 °C. After the reaction mixture was stirred at room temperature for 1 h, the precipitate was collected by filtration and washed with cold THF to obtain the product as a white solid (yield 86%). ^{1}H NMR (400 MHz, Dimethyl sulfoxide-d_6) δ 4.50 (d, J = 16.8 Hz, 2H), 2.13 (d, J = 15.5 Hz, 9H). HRMS (ESI): calculated for $[M]^+$ (cation): m/z 116.06291, found: m/z 116.06339.

Trimethyl phosphorus ylide monomer 2

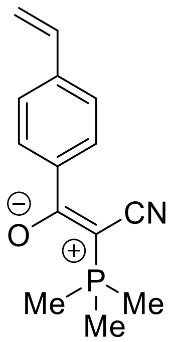

4-vinyl benzoic acid (1.15 g, 7.78 mmol, 1.0 eq) was dissolved in dry CH_2Cl_2 (47 mL) in a flame dried and Argon flashed Schlenk tube and the mixture was stirred. Triethylamine (3.3 mL, 23.3 mmol, 3.0 eq) was added followed by T3P (6.0 mL, 10.1 mmol, 1.3 eq). The mixture was stirred for 15 min and then the phosphonium salt **S2** (1.65 g, 10.9 mmol, 1.4 eq) was added. The reaction mixture was bubbled for 10 min and then stirred at room temperature overnight. It was then diluted with CH_2Cl_2 and it was washed with sat. $NaHCO_3$ (x1), H_2O (x1) and brine (x1). The crude product was dried over Na_2SO_4 and purified via column chromatography on silica gel eluting with CH_2Cl_2/MeOH mixtures (gradient 94:6 to 92:8 by volume) to obtain the product as a light yellow solid (yield 29%). ^{1}H NMR (400 MHz, Chloroform-*d*) δ 7.95 – 7.87 (m, 2H), 7.48 – 7.40 (m, 2H), 6.73 (dd, J = 17.6, 10.9 Hz, 1H), 5.81 (d, J = 17.6, 0.9 Hz, 1H), 5.31 (d, J = 10.9, 0.9 Hz, 1H), 1.88 (d, J = 13.6 Hz, 9H). 13C NMR (101 MHz, Chloroform-*d*) δ 189.75, 189.71, 139.97, 138.10, 138.02, 136.34, 128.04, 125.87, 121.76, 121.60, 115.33, 49.11, 47.88, 11.94, 11.33. ^{31}P NMR (162 MHz, Chloroform-*d*) δ 12.53. HRMS (ESI): calculated for $[M+H]^+$: m/z 246.10478, found: m/z 246.10201.

Benzoic acid triphenyl phosphorus ylide S3

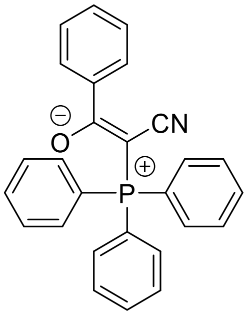

Benzoyl chloride (0.036 mL, 0.306 mmol, 1.0 eq) was dissolved in CH_2Cl_2 (1.6 mL) and the mixture was stirred. Triethylamine (0.130 mL, 0.918 mmol, 3.0 eq) was added and the mixture was stirred for a while and then the phosphonium salt **S1** (0.145 g, 0.429 mmol, 1.4 eq) was added. The reaction mixture was stirred at room temperature overnight. It was then diluted with CH_2Cl_2 and it was washed with sat. $NaHCO_3$ (x1), H_2O (x1) and brine (x1). The crude product was dried over Na_2SO_4 and purified via column chromatography on silica gel eluting with CH_2Cl_2/MeOH mixtures (gradient 95:5 to 9:1 by volume) to obtain the product as a yellow solid (yield 84%). ^{1}H NMR (400 MHz, Chloroform-*d*) δ 8.07 – 7.97 (m, 2H), 7.75 – 7.66 (m, 6H), 7.66 – 7.59 (m, 3H), 7.57 – 7.48 (m, 6H), 7.47 – 7.35 (m, 3H). ^{13}C NMR (101 MHz, Chloroform-*d*) δ 190.18, 139.11, 139.02, 133.73, 133.63, 133.05, 133.02, 130.92, 129.23, 129.10, 128.10, 128.02, 123.93, 123.00, 122.68, 122.52, 49.33, 48.06. ^{31}P NMR (162 MHz, Chloroform-*d*) δ 22.13. HRMS (ESI): calculated for $[M+H]^+$: m/z 406.13608, found: m/z 406.13539.

Benzoic acid trimethyl phosphorus ylide 9

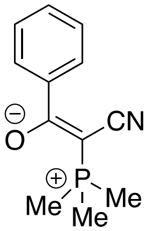

Benzoic acid (316 mg, 2.59 mmol, 1.0 eq) was dissolved in anhydrous CH_2Cl_2 (16 mL). Et_3N (1.1 mL, 7.77 mmol, 3.0 eq) was added to the solution, followed by T3P (3 mL, 3.37 mmol, 1.4 eq). The reaction mixture was saturated with nitrogen for 15 min and then phosphonium salt **S2** (551 mg, 3.63 mmol, 1.4 eq) was added. The resulting mixture was

allowed to stir overnight at room temperature. The mixture was diluted with CH_2Cl_2 and washed with sat. $NaHCO_3$ (x1), H_2O (x1) and brine (x1), dried over Na_2SO_4 and concentrated under reduced pressure. The residue was purified via column chromatography on silica gel eluting with CH_2Cl_2/MeOH mixtures (gradient 100:0 to 20:1 by volume) to obtain the product as a pink solid (5 %). ^{1}H NMR (400 MHz, Chloroform-*d*) δ 7.95 – 7.88 (m, 2H), 7.47 – 7.35 (m, 3H), 1.90 (d, *J* = 12.0 Hz, 9H). ^{13}C NMR (101 MHz, Chloroform-*d*) δ 190.56 (d, *J* = 4.0 Hz), 138.89 (d, *J* = 8.1 Hz), 131.05, 128.19, 127.82, 126.94, 121.75 (d, *J* = 16.1 Hz), 11.81 (d, *J* = 61.6 Hz). ^{31}P NMR (162 MHz, Chloroform-*d*) δ 12.50. MS (ESI) Calculated for $[M+Na]^+$: m/z 242.07, found: m/z 242.30.

3.2 Polymerizations

General Protocol: A flame dried Schlenk tube was purged with Argon and charged with the RAFT agent (2-[[(2-Carboxyethyl)sulfanylthiocarbonyl]-sulfanyl]propanoic acid) and dissolved in anhydrous DMF (unless stated otherwise). The monomers were added under an Argon atmosphere and the solution was stirred. Subsequently, AIBN was added to the solution (in a stock solution), followed by trioxane as internal standard. The solution was degassed for 20 min and a NMR sample for was taken (t = 0 h). The solution was heated to 80 ^{o}C (unless stated otherwise) and the reaction was monitored by ^{31}P and ^{1}H-NMR. After the desired conversion was indicated, the reaction solution was allowed to reach to room temperature and exposed to air. The product was precipitated dropwise in either cold Et_2O or MeOH, re-dissolved in CH_2Cl_2 and subsequently precipitated dropwise in either cold Et_2O or MeOH again. The product was dried under high vacuum overnight.

The molecular weight $M_{n(\mathrm{NMR})}$ was determined *via* ^{1}H-NMR using the alpha-protons adjacent to the trithiocarbonate residue for polymers **3** and **4**. For polymers **5**, **6** and **7** the acidic protons of the carboxylic acid were used (example in Figure S6a and b).

a)

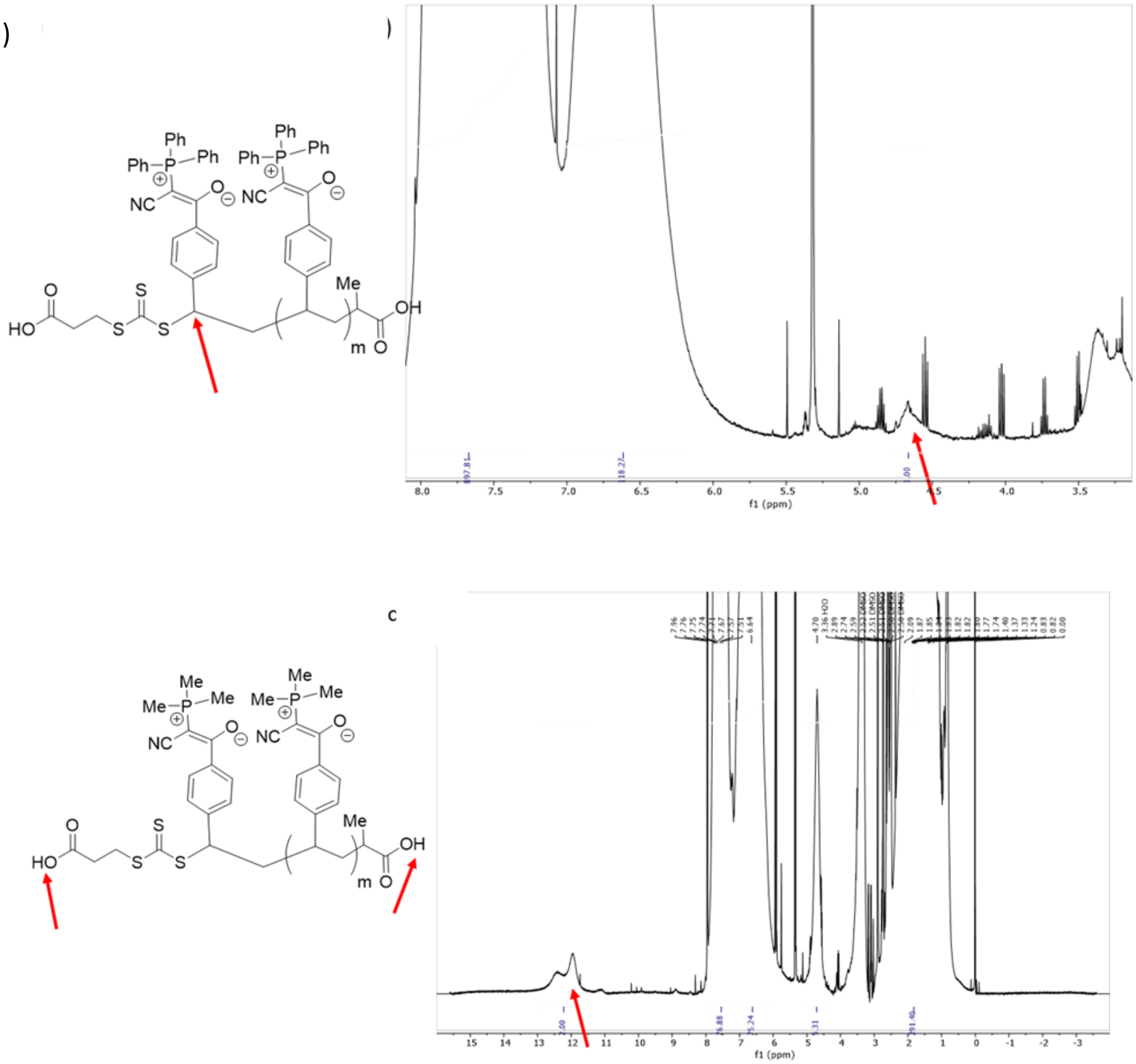

Figure S8. Reference protons used for the calculation of the M_n and ^{1}H NMR of the polymers, a) The protons used as reference for the triphenyl phosphorus ylide polymers **3** and **4**, b) The protons used as reference for the trimethyl phosphorus ylide polymers **5**, **6** and **7**.

PS-*co*-P(TPPY) 3

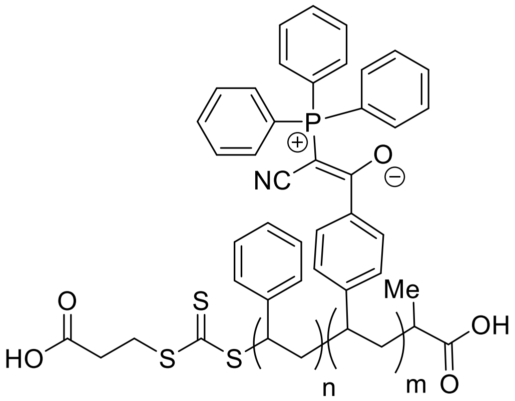

The general procedure for the polymer synthesis was followed with RAFT agent (8.0 mg, 0.0316 mmol, 1.0 eq), triphenyl phosphorus ylide monomer **1** (0.300 g, 0.695 mmol, 22 eq), styrene (0.0724 g, 0.695 mmol, 22 eq) and AIBN (1.8 mg, 0.0111 mmol, 0.35 eq) in dimethyl sulfoxide (1.0 mL). The product was precipitated dropwise in cold MeOH, re-dissolved in CH_2Cl_2 and subsequently precipitated dropwise in cold MeOH again. The product was isolated as a yellow solid (96.3 mg).

GPC (N-Methyl-2-pyrrolidone, polystyrene calibration): M_n=7.5 × 103 g/mol, M_w/M_n = 1.13
^{1}H-NMR (500 MHz, Chloroform-*d*) δ(ppm): 8.05–6.18 (br. m, arom. C-H of PS and P(SY)), 2.40–0.75 (br. m, backbone). Mn (NMR) =9.1 × 10^3 g/mol. F_{PY} = 0.48

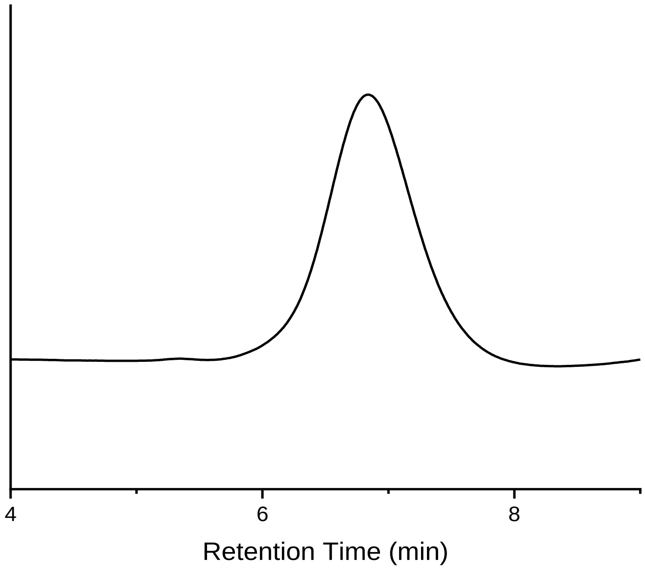

P(TPPY) 4

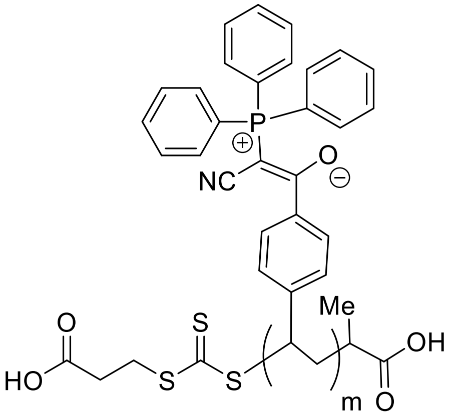

The general procedure for the polymer synthesis was followed with RAFT agent (9.5 mg, 0.0374 mmol, 1.0 eq), triphenyl phosphorus ylide monomer **1** (0.500 g, 1.16 mmol, 31 eq) and AIBN (2.2 mg, 0.0131 mmol, 0.35 eq) in dimethyl sulfoxide (0.78 mL). The product was precipitated dropwise in cold MeOH, re-dissolved in CH_2Cl_2 and subsequently precipitated dropwise in cold MeOH again. The product was isolated as a yellow solid (127 mg).

GPC (N-Methyl-2-pyrrolidone, polystyrene calibration): M_n=6.3 × 103 g/mol, M_w/M_n = 1.15
^{1}H-NMR (500 MHz, Chloroform-*d*) δ(ppm): 8.06–6.19 (br. m, arom. C-H of PS and P(SY)), 2.45–0.66 (br. m, backbone). M_n (NMR) =5.2 × 10^3 g/mol.

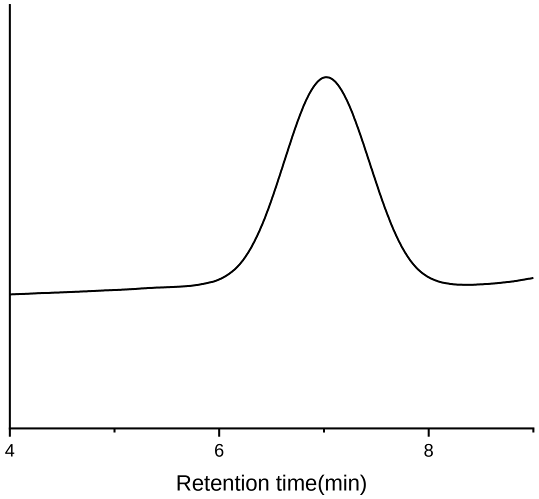

PS-co-P(TMPY) 5

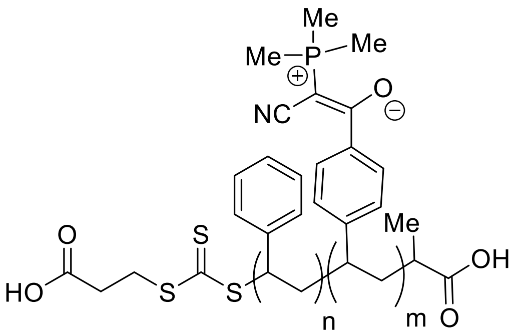

The general procedure for the polymer synthesis was followed with RAFT agent (5.5 mg, 0.0214 mmol, 1.0 eq), trimethyl phosphorus ylide monomer **2** (0.200 g, 0.815 mmol, 38 eq), styrene (0.100 mL, 0.815 mmol, 38 eq) and AIBN (1.23 mg, 0.00749 mmol, 0.35 eq) in dry dimethyl formamide (1.1 mL). The polymerization was performed at 70 ^{o}C. The product was precipitated dropwise in cold Et_2O, re-dissolved in CH_2Cl_2 and subsequently precipitated dropwise in cold Et_2O again. The product was isolated as a yellow solid (146 mg).

GPC (Dimethylacetamide, polystyrene calibration): M_n=6.9 × 103 g/mol, M_w/M_n = 1.19

^{1}H-NMR (500 MHz, dimethyl sulfoxide-*d*$_6$) δ (ppm): 7.90–6.20 (br. m, arom. C-H of PS and P(SY)), 2.00–1.70 (br. s, P(CH3)3), 2.35–0.76 (br. m, backbone). M_n (NMR) = 6.6 × 10^3 g/mol. F_{PY} = 0.43

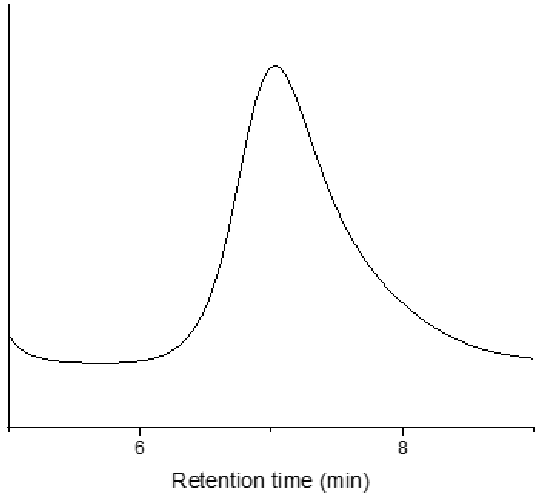

P(TMPY) 6

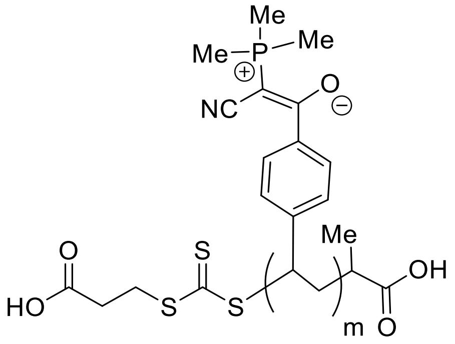

The general procedure for the polymer synthesis was followed with RAFT agent (7.8 mg, 0.0308 mmol, 1.0 eq), trimethyl phosphorus ylide monomer **2** (0.400 g, 1.63 mmol, 53 eq) and AIBN (1.5 mg, 0.00924 mmol, 0.30 eq) in dry dimethyl formamide (1.1 mL). The product was precipitated dropwise in cold Et_2O, re-dissolved in CH_2Cl_2/MeOH 1:1 and subsequently precipitated dropwise in cold Et_2O again. The product was isolated as a yellow solid (162 mg).

GPC (N-Methyl-2-pyrrolidone, polystyrene calibration): M_n=5.4 × 103 g/mol, M_w/M_n = 1.10
^{1}H-NMR (500 MHz, dimethyl sulfoxide-d_6) δ(ppm): 7.90–6.13 (br. m, arom. C-H of PS and P(SY)), 2.00–1.67 (br. s, P(CH3)3), 2.34–0.74 (br. m, backbone). M_n (NMR) = 9.1 × 10^3 g/mol.

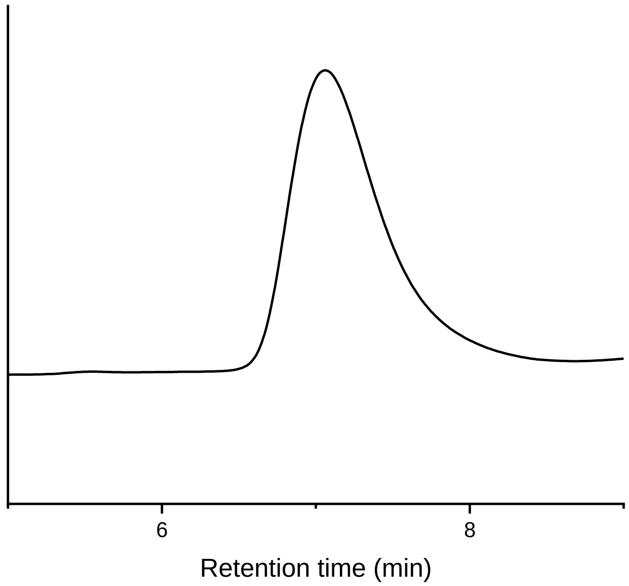

P(TMPY) 7

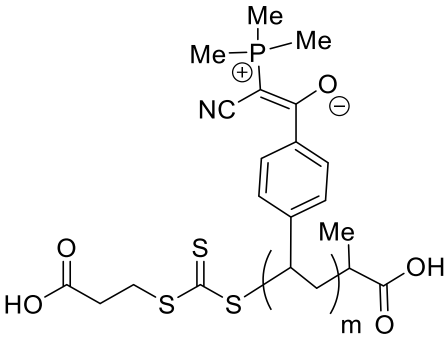

The general procedure for the polymer synthesis was followed with RAFT agent (5.9 mg, 0.0230 mmol, 1.0 eq), trimethyl phosphorus ylide monomer **2** (0.300 g, 1.22 mmol, 53eq) and AIBN (1.3 mg, 0.00805 mmol, 0.35 eq) in dry DMF (0.82 mL). The polymerization was performed at 70 ^{o}C. The product was precipitated dropwise in cold Et_2O, re-dissolved in CH2Cl2/MeOH 1:1 and subsequently precipitated dropwise in cold Et_2O again. The product was isolated as a yellow solid (126 mg).

GPC (Dimethylacetamide, polystyrene calibration): M_n=3.1 × 103 g/mol, M_w/M_n = 1.14

^{1}H-NMR (500 MHz, dimethyl sulfoxide-*d_6*) δ(ppm): 7.88–6.09 (br. m, arom. C-H of PS and P(SY)), 1.96–1.66 (br. s, P(CH3)3), 2.30–0.76 (br. m, backbone). M_n (NMR) = 5.6 × 10^3 g/mol.

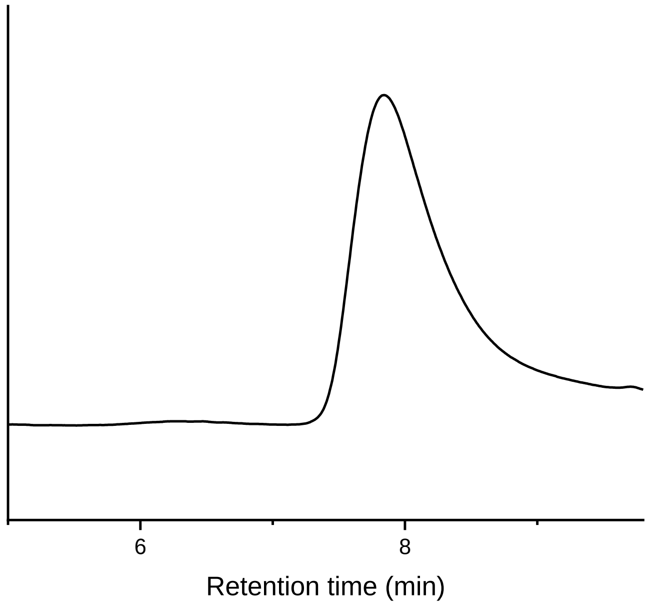

Synthesis of Poly[[2-(Methacryloyloxy)ethyl]dimethyl-(3-sulfopropyl)ammonium hydroxide] P(DMAPS) 8

PSBMA homopolymer was synthesized according to previously reported.[8] Briefly, in a microwave vial previously dried at 130 °C overnight, SBMA (120 mg, 0.43 mmol), ACVA (1 mg, 0.003 mmol), CPBD-COOH (4 mg, 0.001 mmol) and Trioxane (64 mg, 0.71 mmol) were dissolved in 1.43 mL NaCl solution (0.5 M) to result in an initial [monomer]:[CTA]:[initiator] ratio of [30]:[1]:[0.25]. The pH value of the solution was adjusted to ~7 by the addition of aq. NaOH (1M). The vial was capped and degassed with Argon for 40 minutes. The reaction mixture was placed in a metallic jacket at 65 °C for 18 h. Polymerization was stopped by cooling down in liquid N_2 and exposing to air. The product was purified by dialysis against deionized water for 2 days (MWCO 3.5 kDa). The product was isolated by lyophilization to yield a pink polymer.

^{1}H-NMR (400 MHz, D_2O,): δ= 0.7-2.14 (m, 117 H, (CH_3, CH_2) of CTA, (CH_3, CH_2) of polymeric backbone), 2.23 (s, 44 H, CH_2), 2.94 (s, 44 H, CH_2), 3.19 (s, 132 H, CH_3), 3.55(s, 44 H, CH_2), 3.76 (s, 44 H, CH_2), 4.45 (s, 44 H, CH_2). M_n (NMR) = 6.4 × 10^3 g/mol.

4. Trimethyl phosphorus ylide 2 monomer oxidation during polymerization

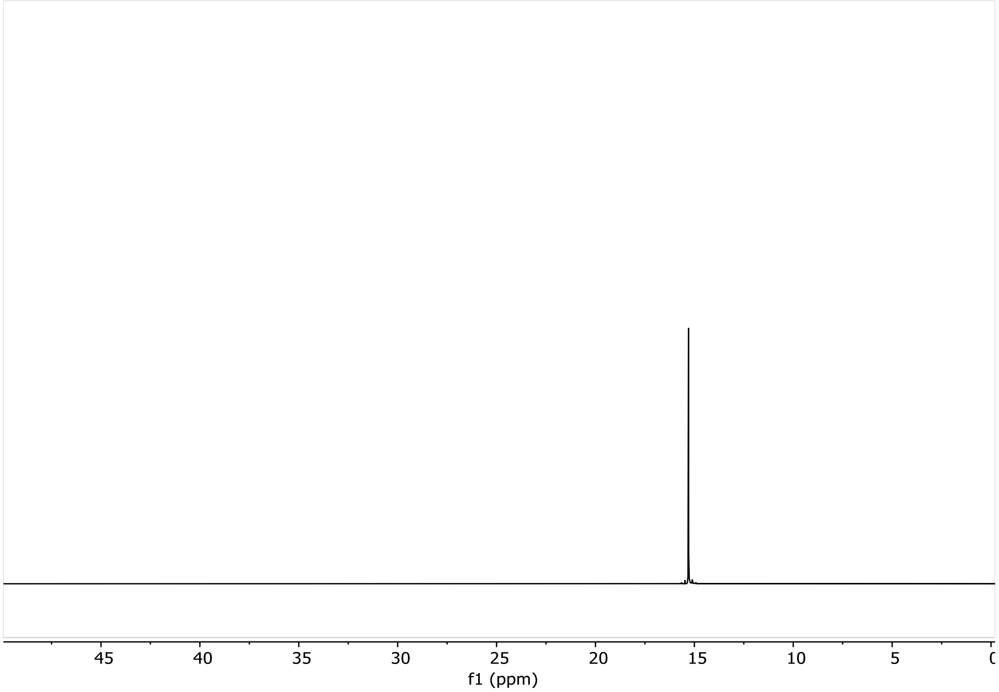

Figure S9. ^{31}P NMR at t_0 of the polymerization of the P(TMPY) **7**.

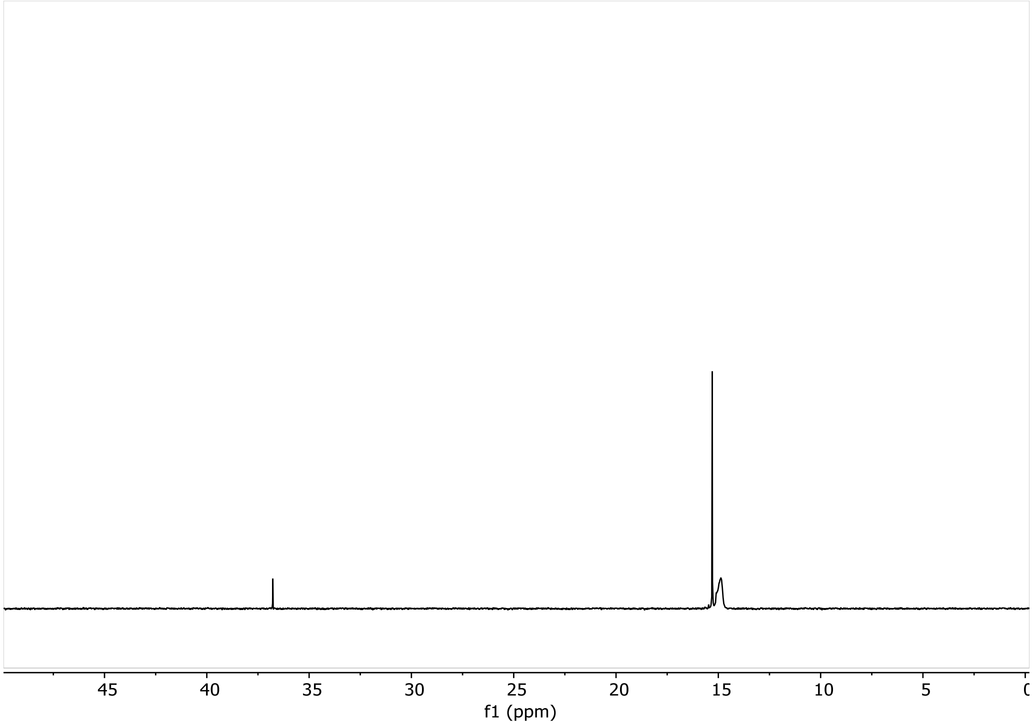

Figure S10. ^{31}P NMR at t_{final} (9 h) of the polymerization of the P(TMPY) **7**. According to NMR, the percentage of monomer oxidation was found to be overall 3.0%.

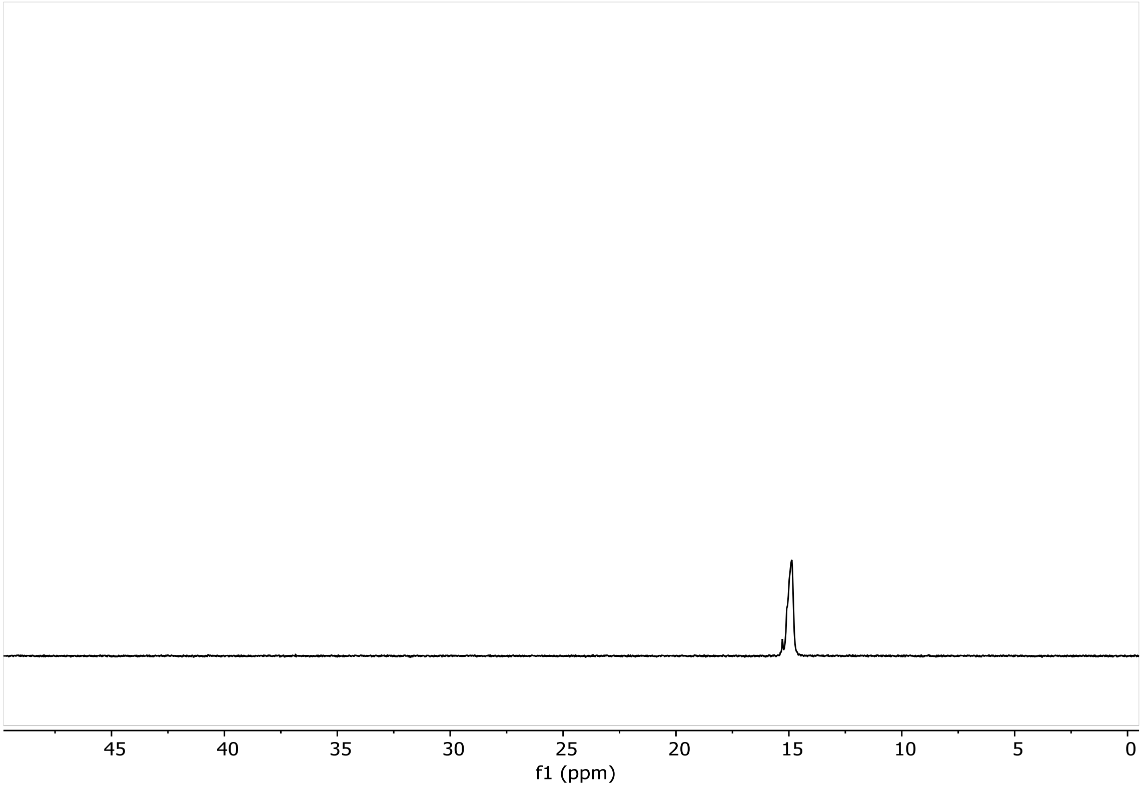

Figure S11. ^{31}P NMR of the P(TMPY) **7**. No oxidation peak is observed.

4. Surface energy – Contact angle

The contact angle and surface energy were measured according to a literature protocol.[9] The contact angle of the polymers was measured by spreading the polymers on a glass surface. Glass surfaces are polar and therefore it is easier for the polymers to spread. Before using them, the glass surfaces were cleaned using 2% Hellmanex solution and sonicated for 10 min with heating. Then they were thoroughly rinsed with demi water and dried well.

The following polymer solutions were prepared:

1. 3 mg of the PS-co-P(TPPY) **3** in 250 μL of dichloromethane
2. 3 mg of the P(TPPY) **4** in 250 μL of dichloromethane
3. 3 mg of the PS-co-P(TMPY) **5** in 250 μL of dichloromethane/MeOH (1:1)
4. 3 mg of the P(TMPY) **7** in 250 μL of dichloromethane/MeOH (1:1)

10 μL of each solution were spread on different glass surfaces, using a pipette and performing a circular movement in order to create a thin layer of evenly spread solution. Then, solutions 1 and 2 were put in the oven at 65 ºC for 5 min while the rest for 15 min

For the contact angle measurement, MilliQ water, glycerol or diidomethane (5 μL) was placed on the glass coated polymer surface and then snapshots were taken using optical microscopy. The images were then analyzed using ImageJ program to determine the contact angles. Each measurement was performed twice.

The surface energy was calculated using the acid-base Van Oss method.[10] For this study, only the homopolymer P(TMPY) was used.

$$\mathbf{1})\ 0.5\,\gamma_L\,(1+cos\Theta) = \sqrt{\gamma_S^{LW}\gamma_L^{LW}} + \sqrt{\gamma_S^{+}\gamma_L^{-}} + \sqrt{\gamma_S^{-}\gamma_L^{+}}$$

$$\mathbf{2})\ \gamma_S = \gamma_S^{LW} + 2\sqrt{\gamma_S^{+}\gamma_S^{-}}$$

Θ: contact angle; γ_S: (total) surface energy of a polymer; γ_{LW}: the Liftshitz/van der Waals (dispersive) component; γ^+: Lewis acid component; γ^-: Lewis-base component; γ_L: surface tension of the liquid; mN/m (or mJ/cm2); L = liquid, S = solid.

Table S3. The values of γ_L^{LW}, γ_L^+, γ_L^- and γ_L for water, glycerol and diidomethane found from literature.[11,12]

	γ_L (mN/m^2)	γ_L^{LW} (mN/m^2)	γ_L^+ (mN/m^2)	γ_L^- (mN/m^2)
**Water (MilliQ)**	72.8	21.8	25.5	25.5
**Glycerol**	64.0	34.0	3.92	57.4
**Diiodomethane**	50.8	50.8	0	0

To find γ_S^{LW}, γ_S^+ and γ_S^-, equation **1** was solved using the contact angles measured and the constants of table **1**. Then γ_S was calculated using equation **2**.

5. NMR Spectra

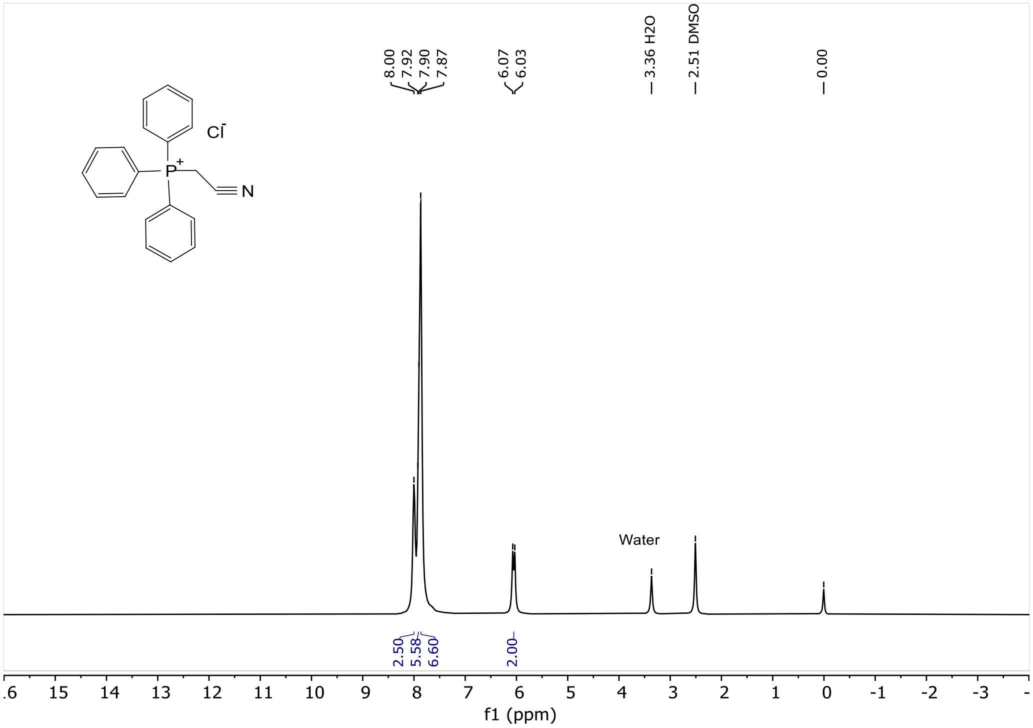

Figure S12: ^{1}H NMR spectrum of the triphenyl phosphonium salt **S1**.

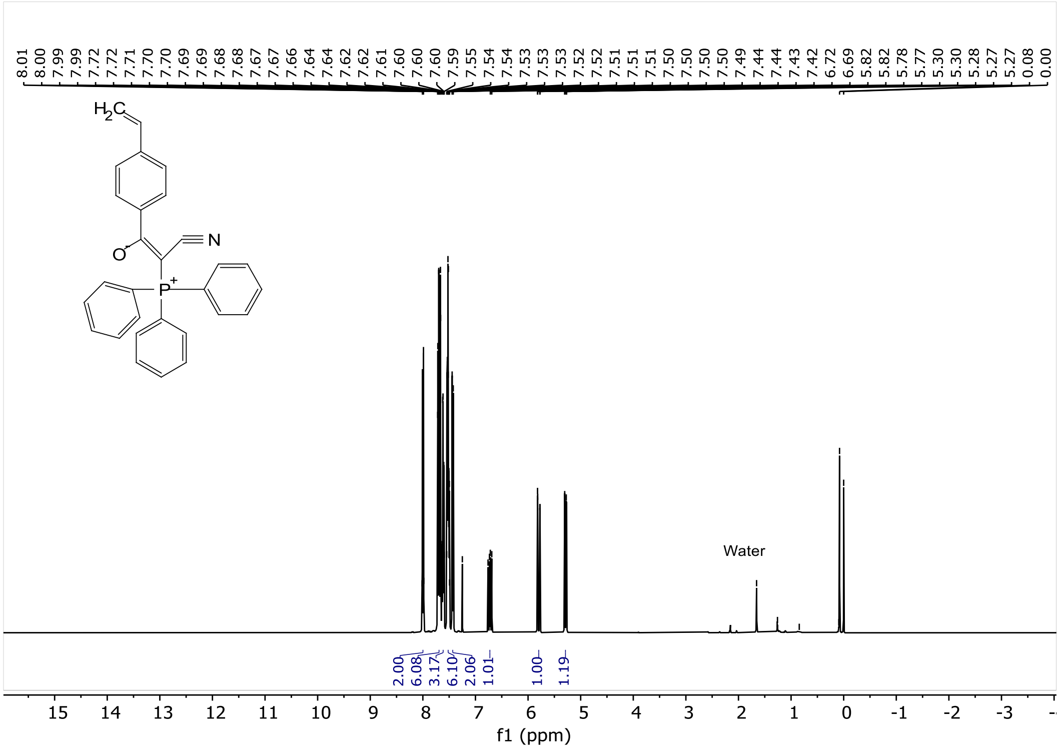

Figure S13: ^{1}H NMR spectrum of the triphenyl phosphorus ylide monomer **1**.

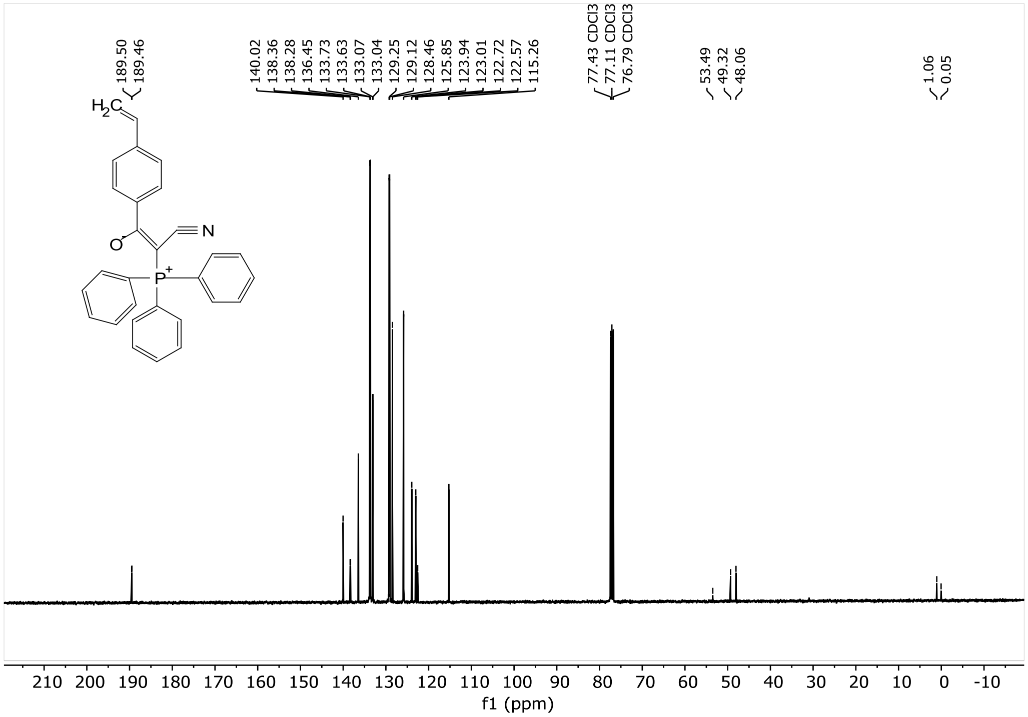

Figure S14: ^{13}C NMR spectrum of the triphenyl phosphorus ylide monomer **1**.

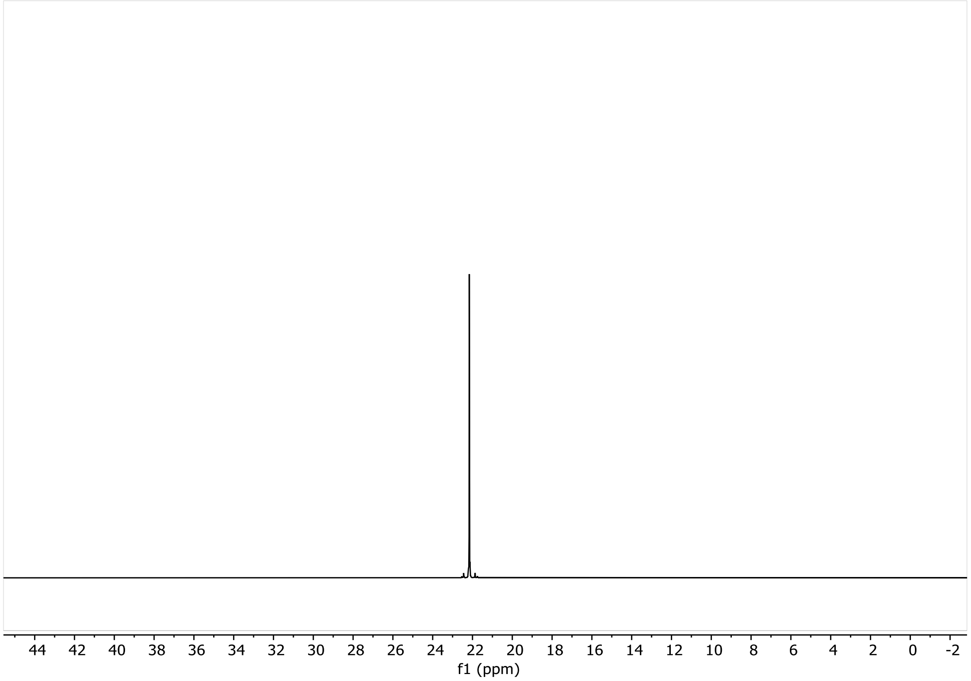

Figure S15: ^{31}P NMR spectrum of the triphenyl phosphorus ylide monomer **1**.

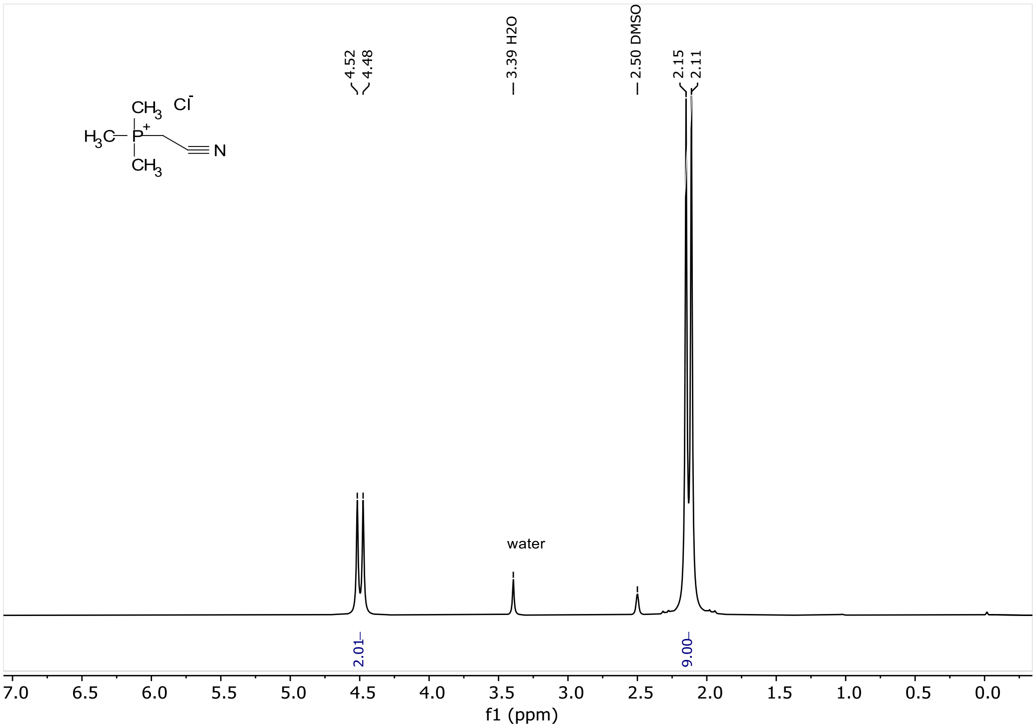

Figure S16. ^{1}H NMR spectrum of the trimethyl phosphonium salt **S2**.

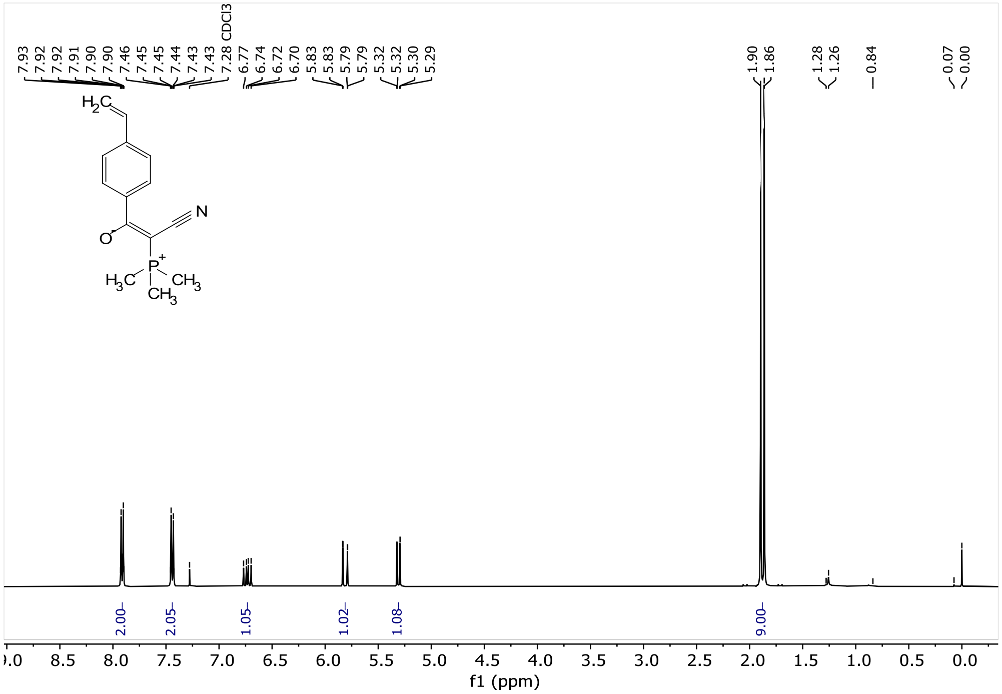

Figure S17. ^{1}H NMR spectrum of the trimethyl phosphor ylide monomer **2.**

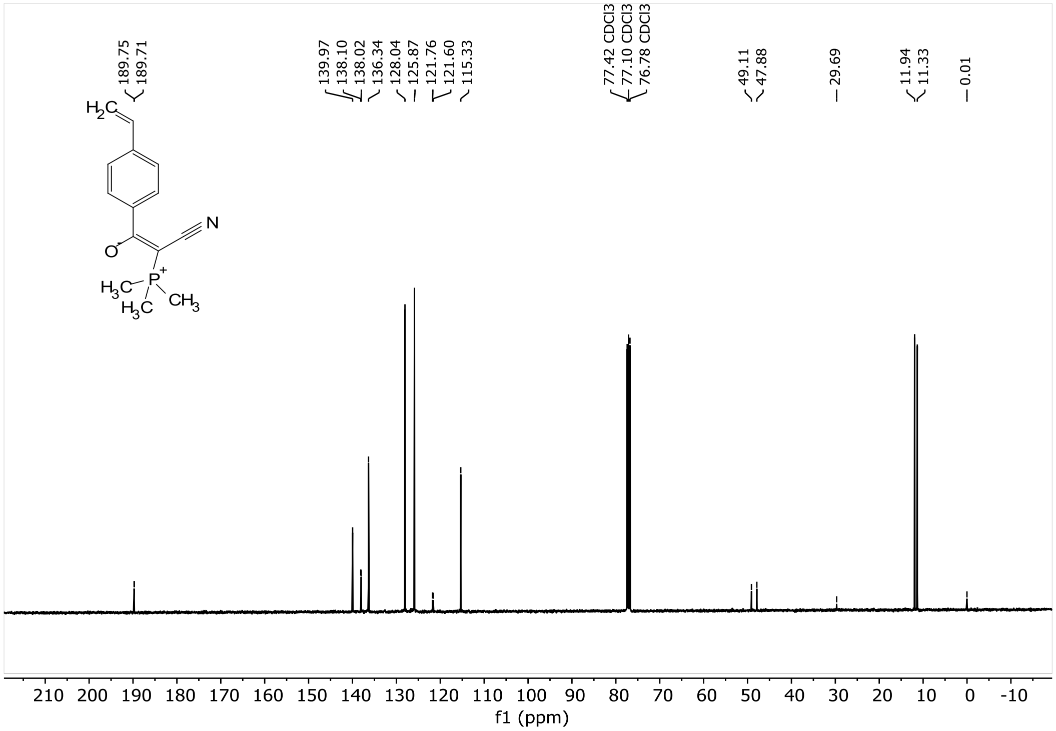

Figure S18. ^{13}C NMR spectrum of the trimethyl phosphorus ylide monomer **2**.

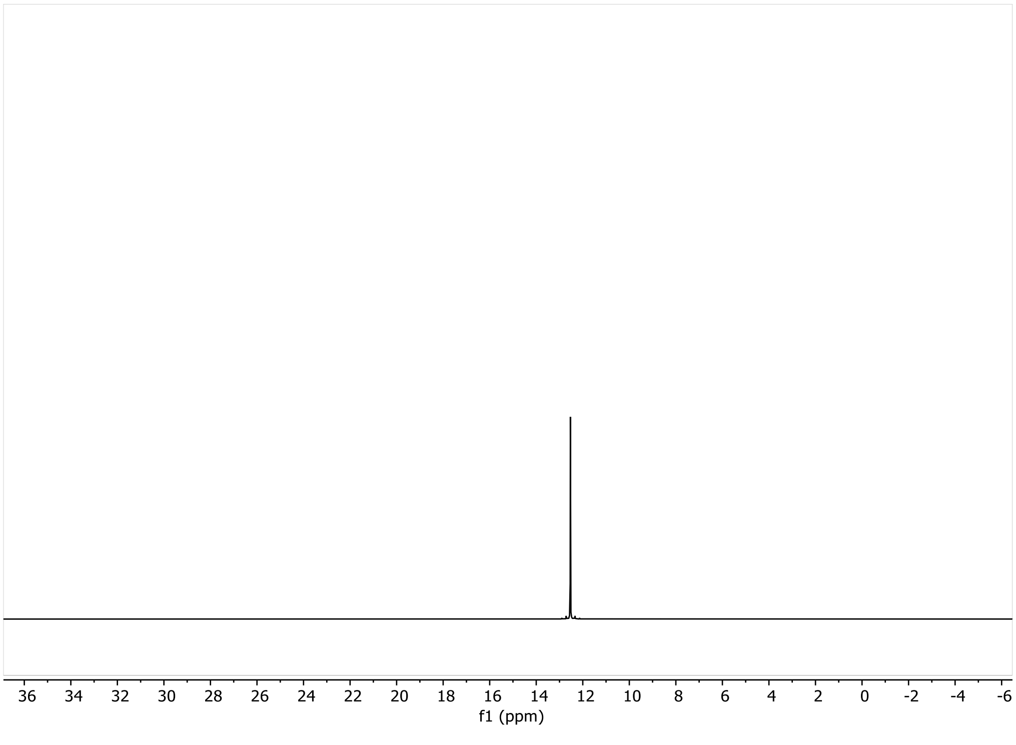

Figure S19. ^{31}P NMR spectrum of the trimethyl phosphorus ylide monomer **2**.

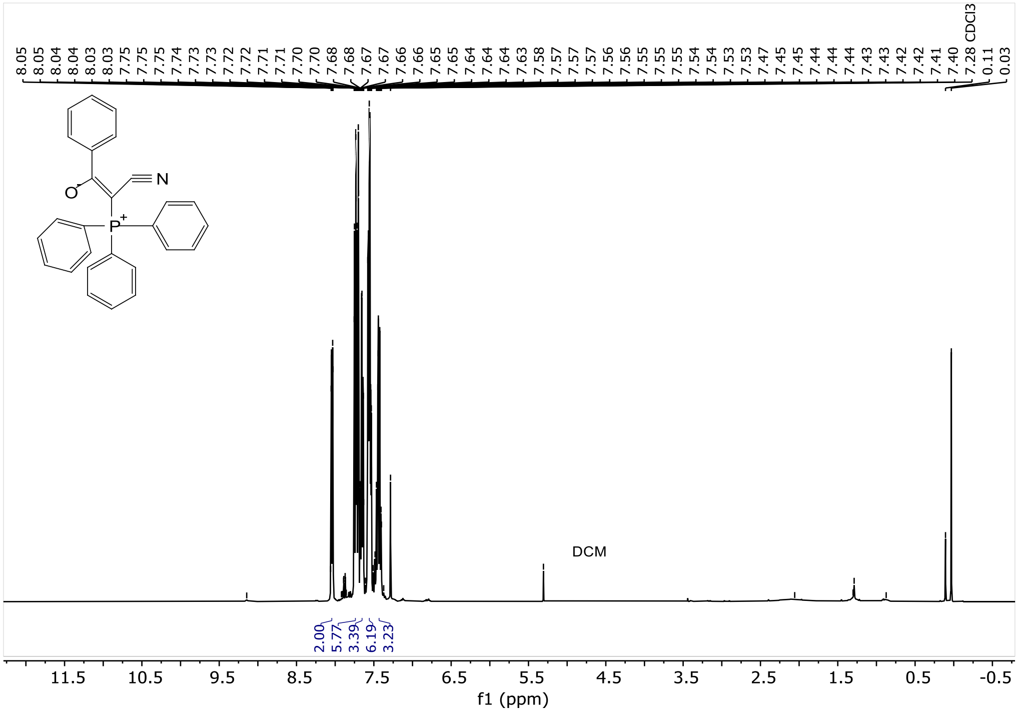

Figure S20. ^{1}H NMR spectrum of the benzoic acid triphenyl phosphor ylide **S3**.

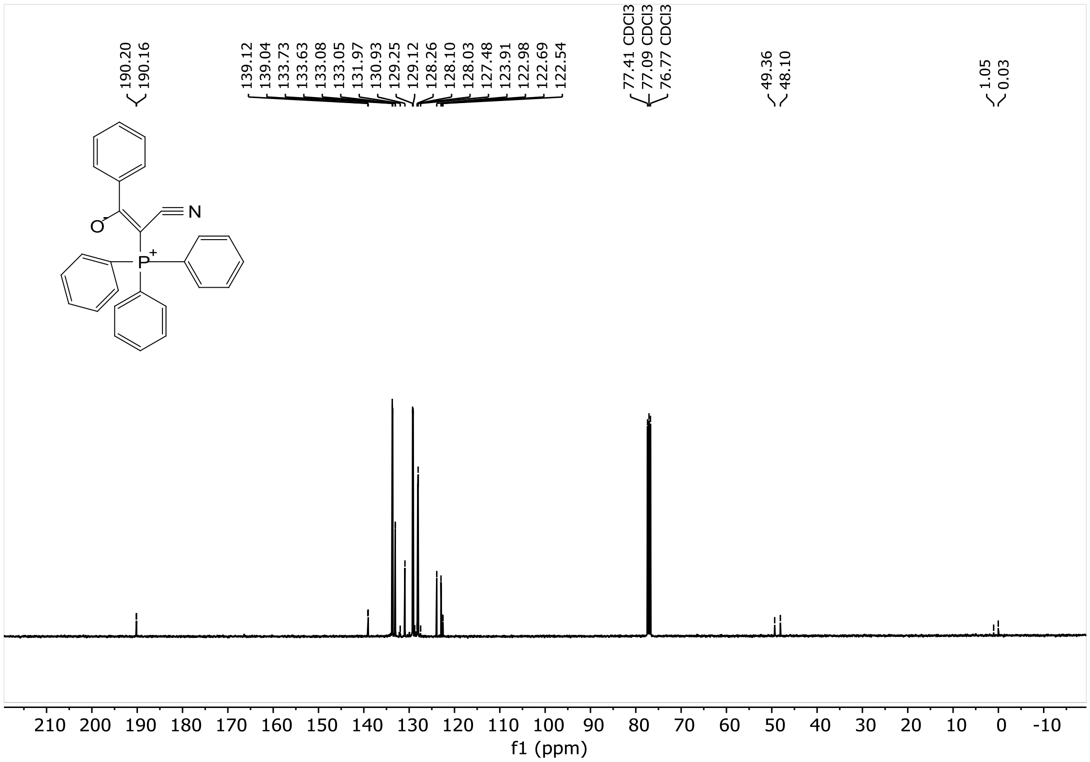

Figure S21. ^{13}C NMR spectrum of the benzoic acid triphenyl phosphorus ylide **S3**.

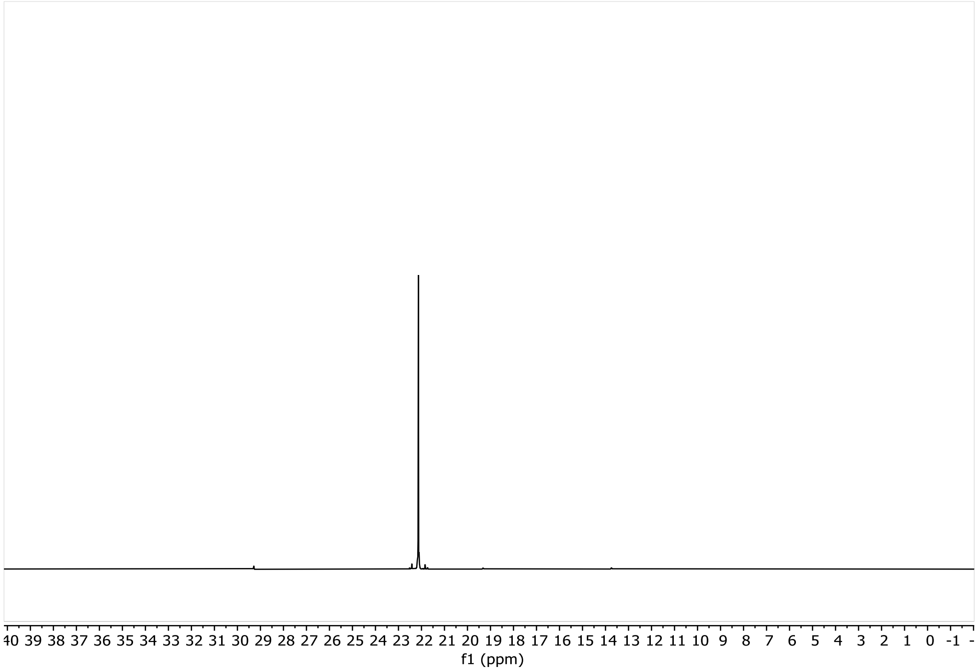

Figure S22. ^{31}P NMR spectrum of the benzoic acid triphenyl phosphorus ylide **S3**.

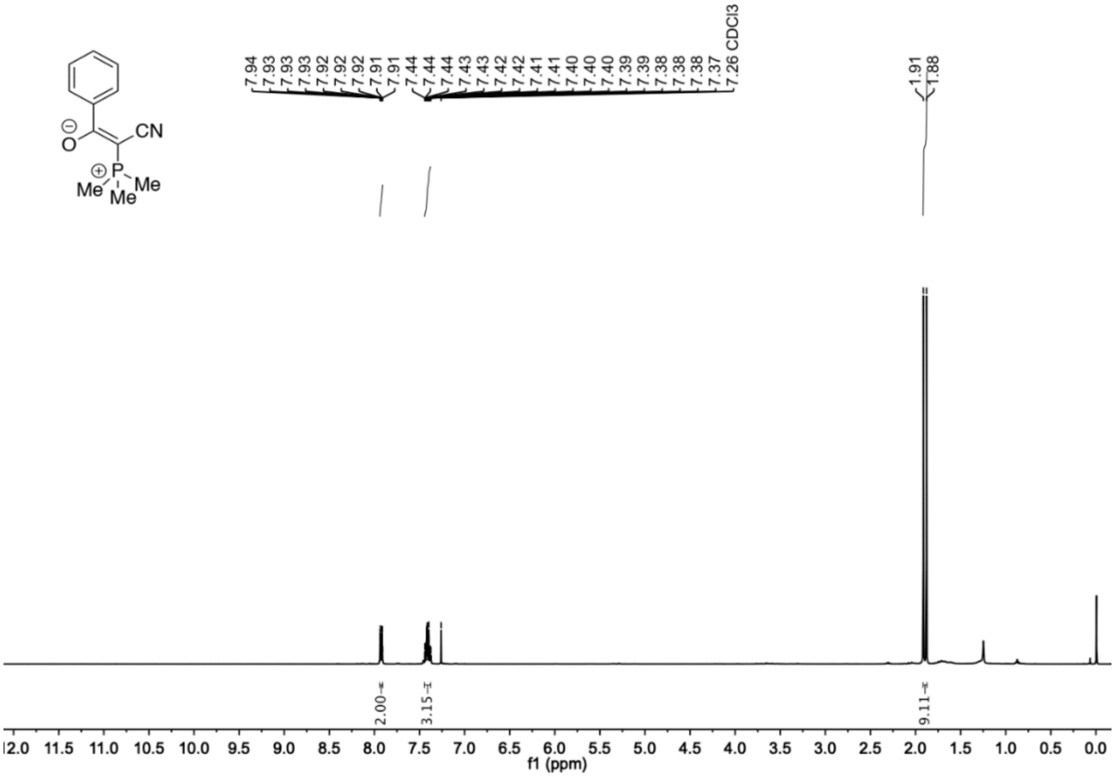

Figure S23. ^{1}H NMR of benzoic acid trimethyl phosphorus ylide **9**.

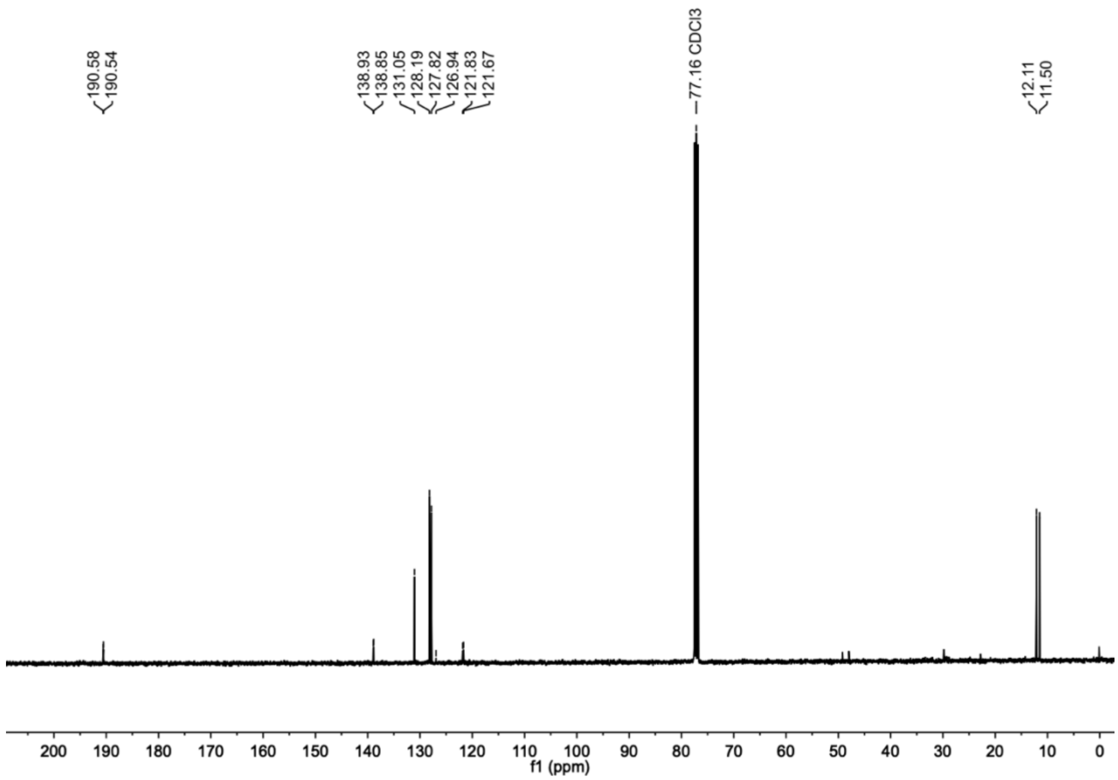

Figure S24. ^{13}C NMR of benzoic acid trimethyl phosphorus ylide **9**.

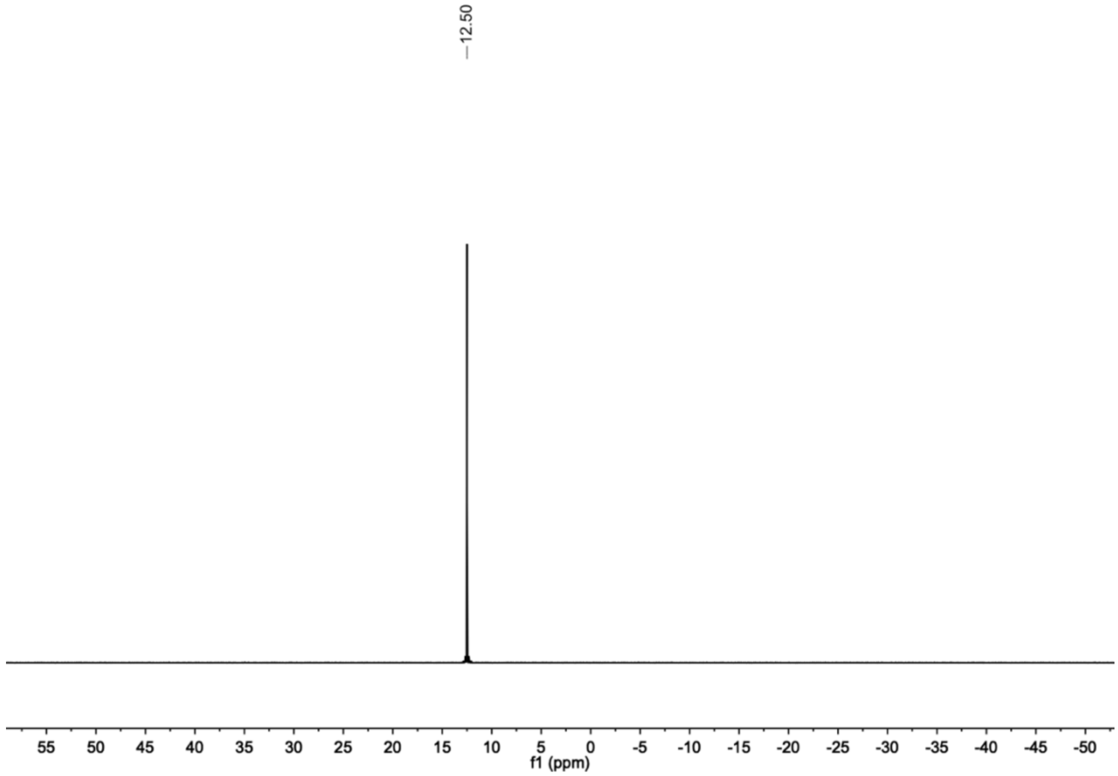

Figure S25. ^{31}P NMR of benzoic acid trimethyl phosphorus ylide **9**.

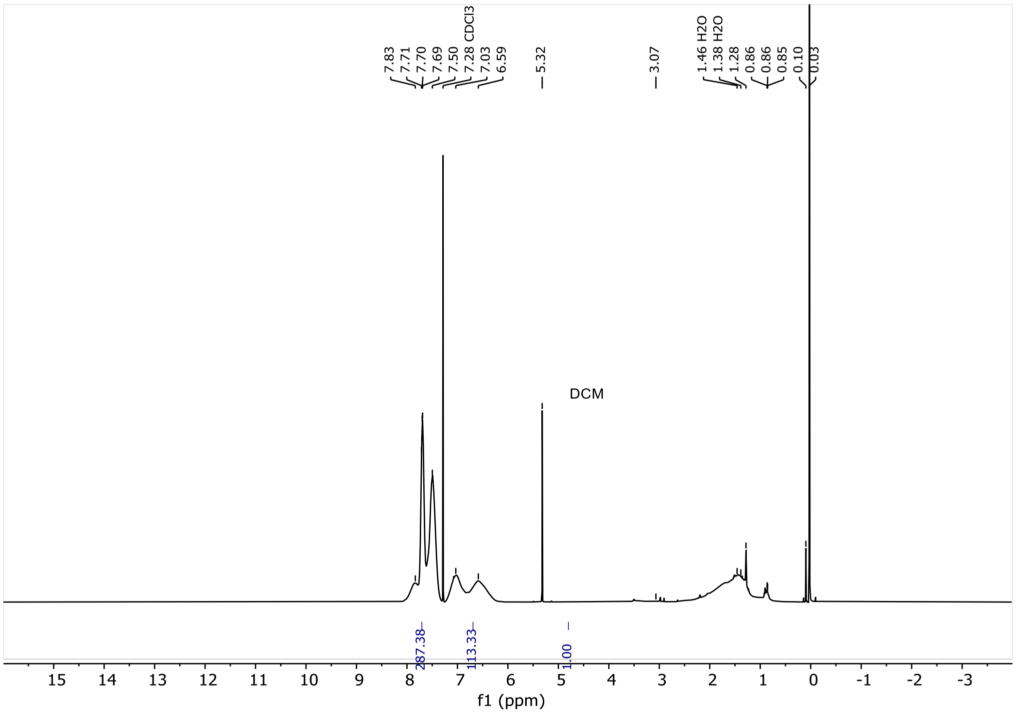

Figure S26. ^{1}H NMR of the PS-*co*-P(TPPY) **3**.

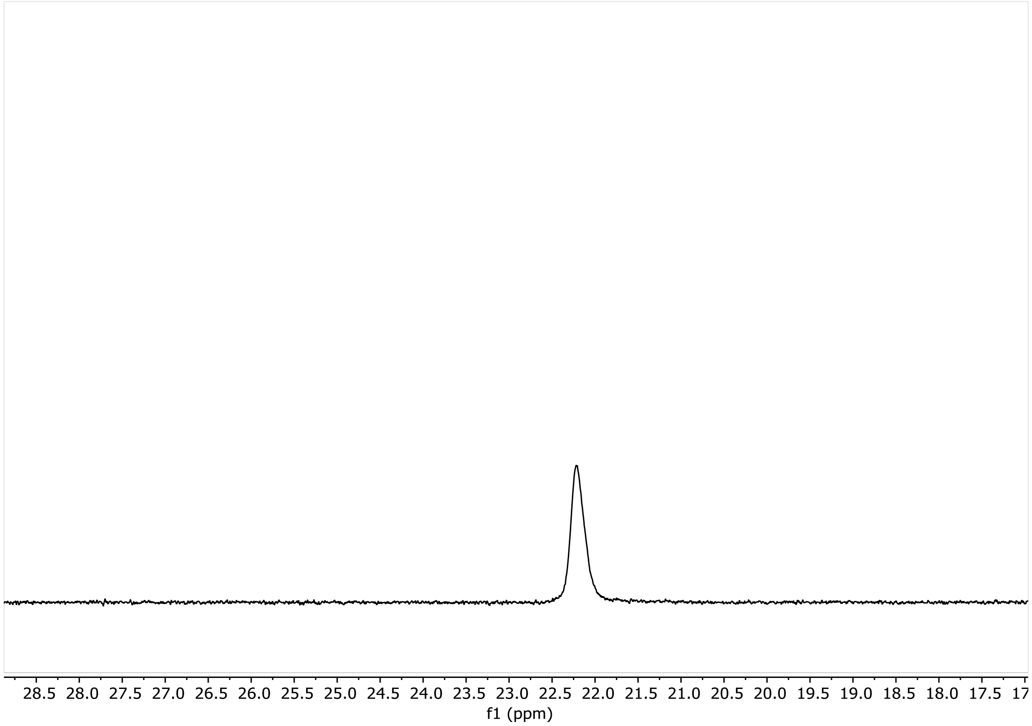

Figure S27. ^{31}P NMR of the PS-*co*-P(TPPY) **3**.

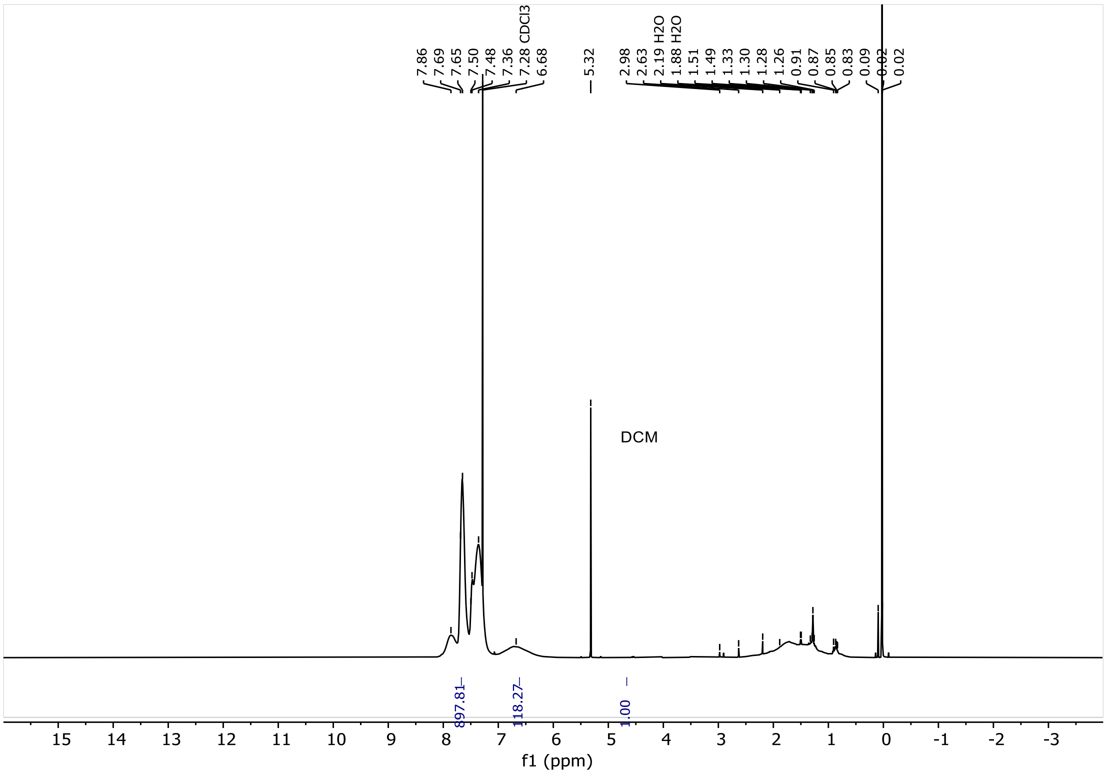

Figure S28. ^{1}H NMR of the P(TPPY) **4**.

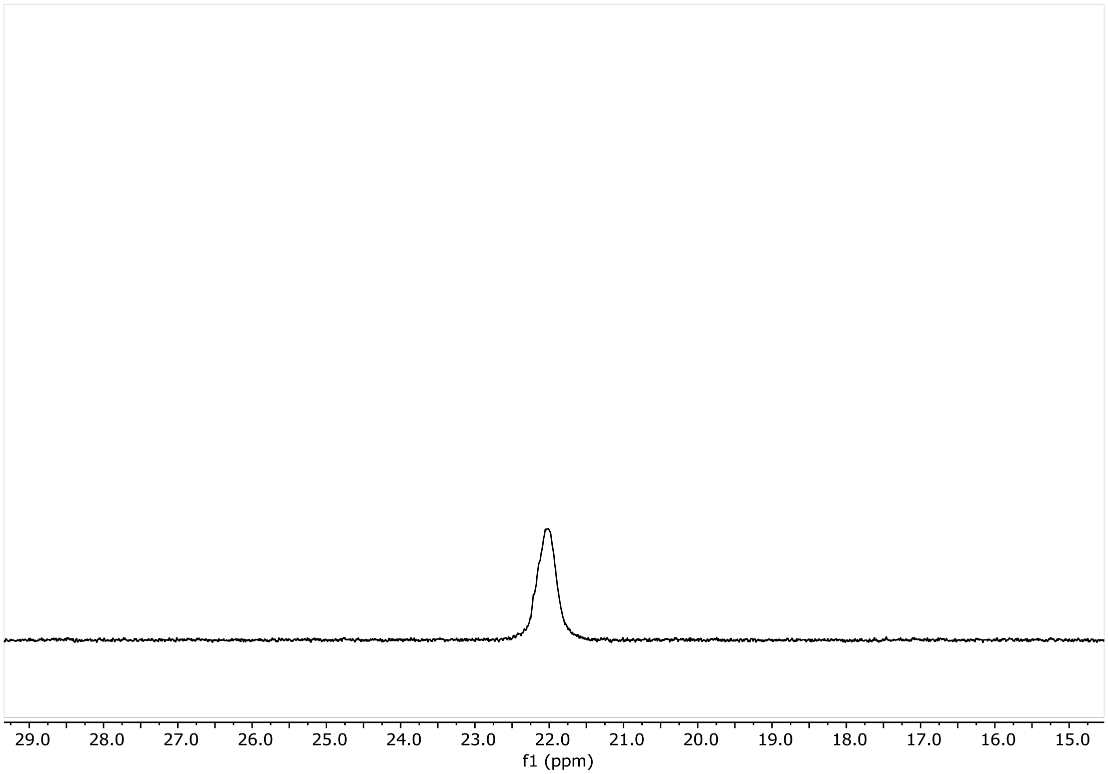

Figure S29. ^{31}P NMR of the P(TPPY) **4**.

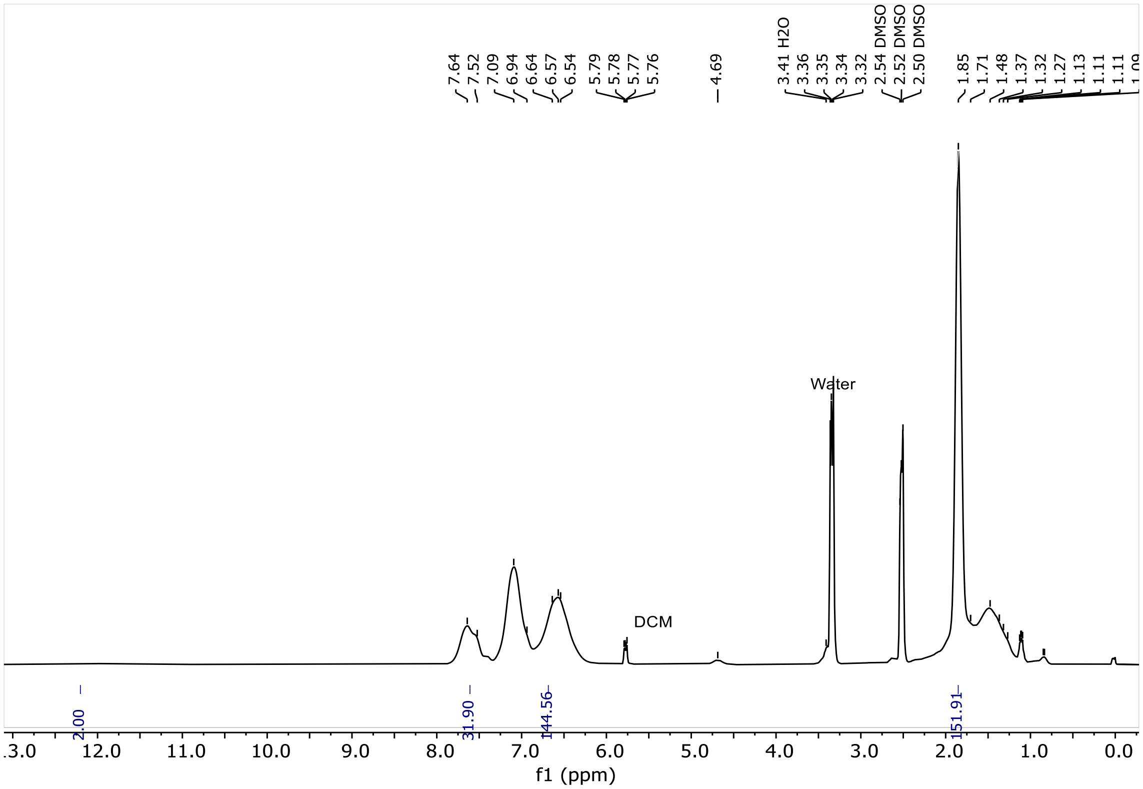

Figure S30. ^{1}H NMR of the PS-*co*-P(TMPY) **5**.

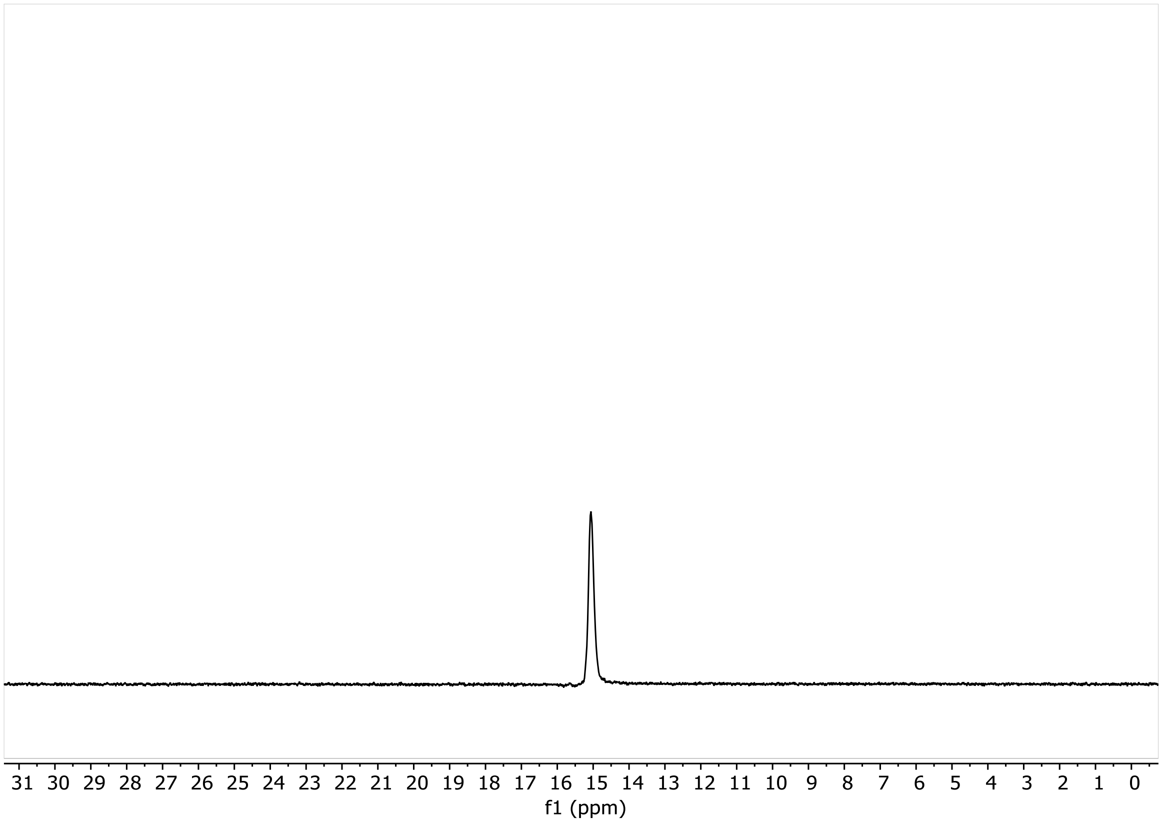

Figure S31. ^{31}P NMR of the PS-*co*-P(TMPY) **5**.

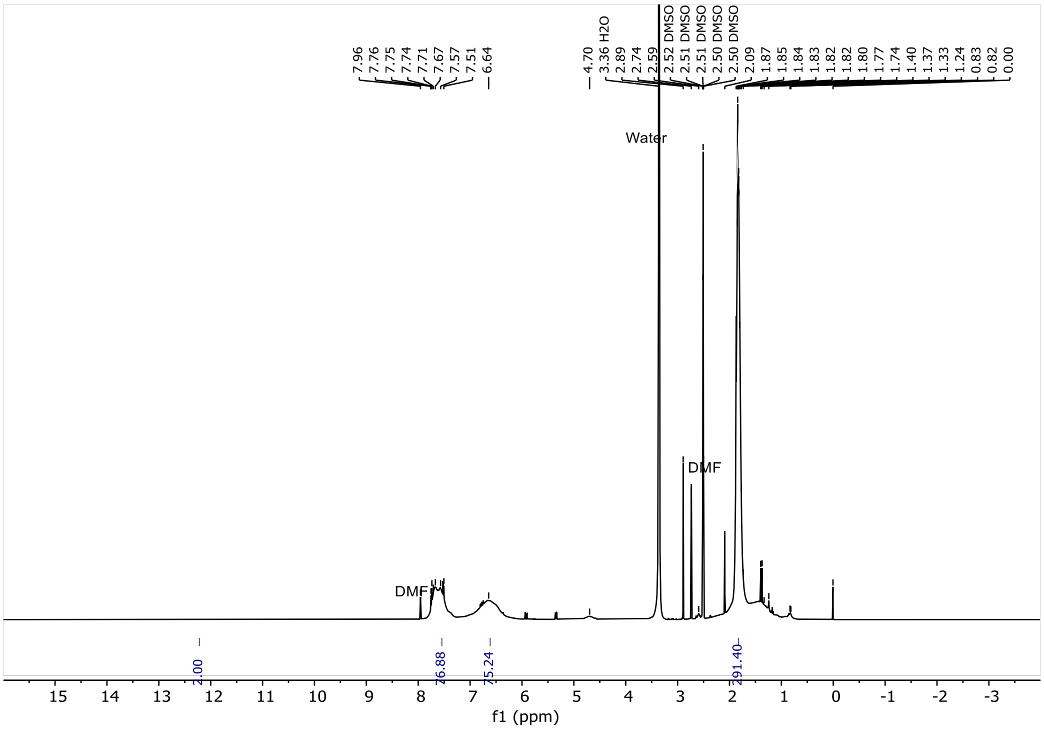

Figure S32. ^{1}H NMR of the P(TMPY) **6**.

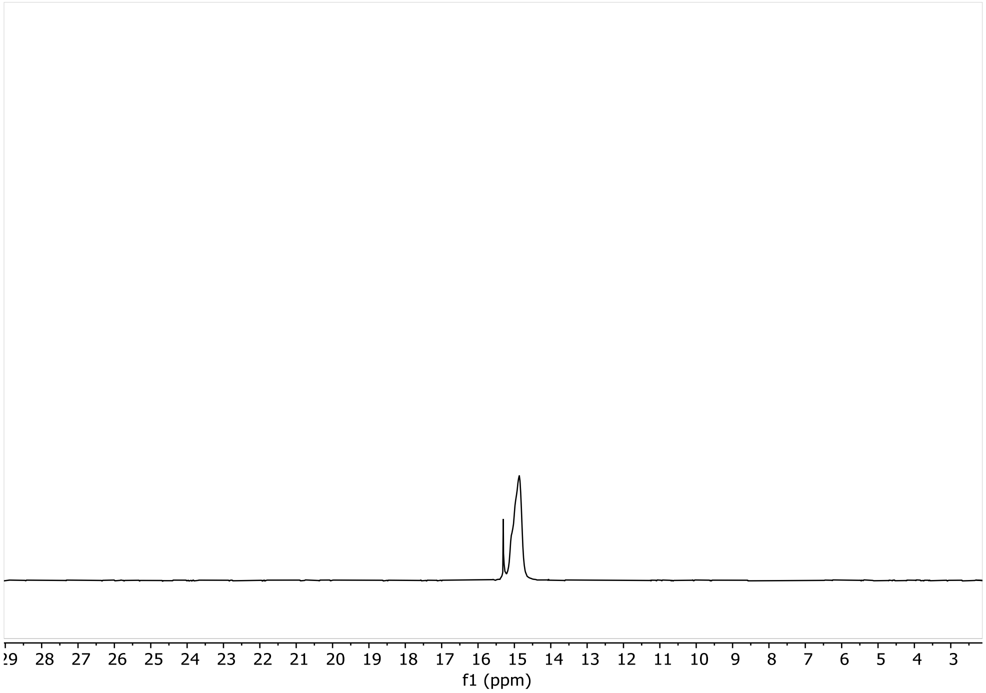

Figure S33. ^{31}P NMR of the P(TMPY) **6**.

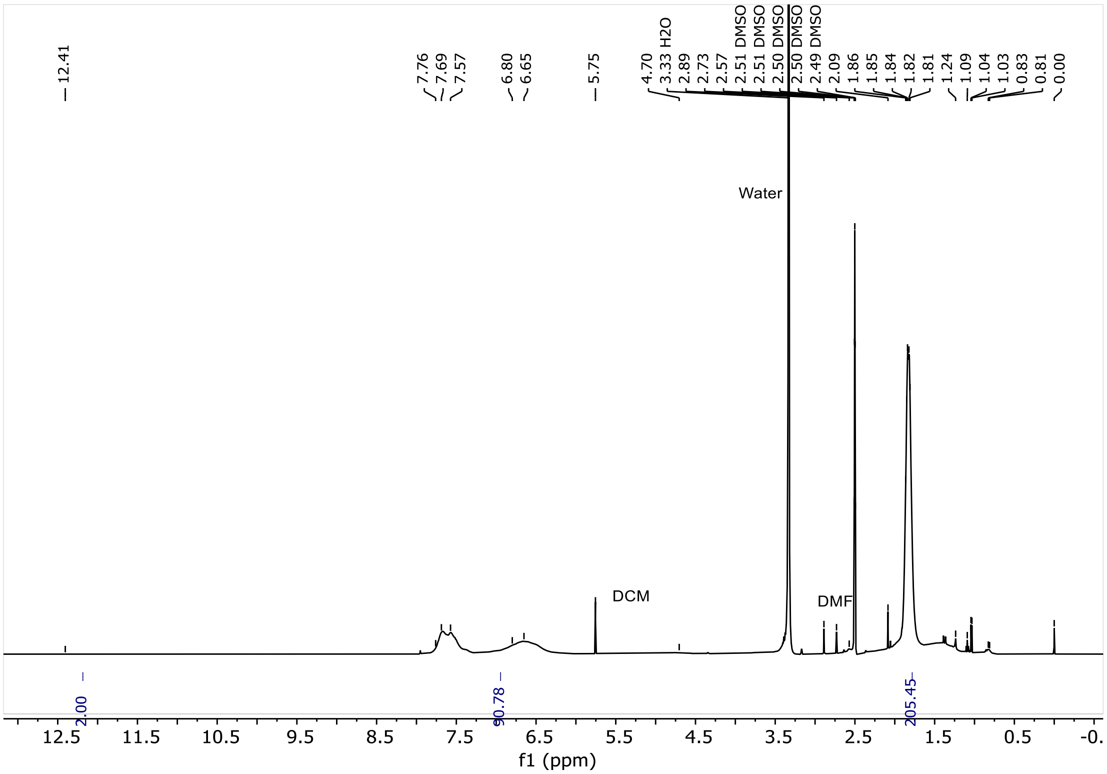

Figure S34. ¹H NMR of the P(TMPY) **7**.

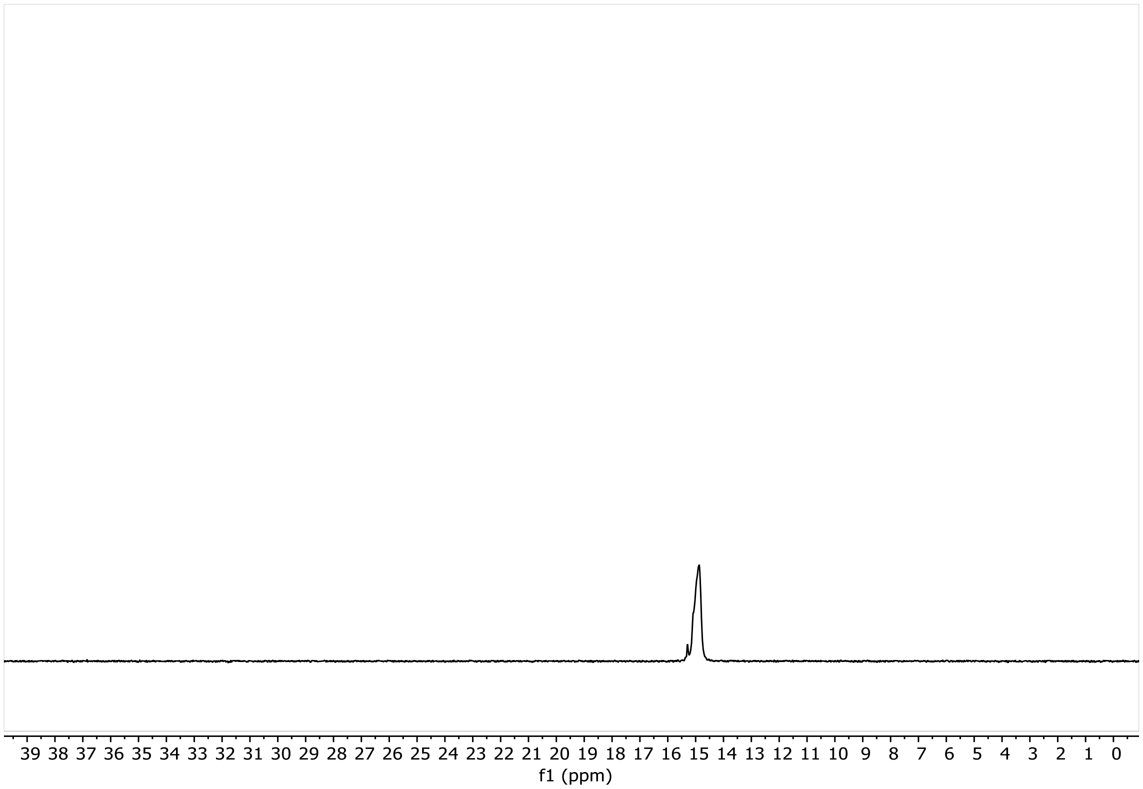

Figure S35. ³¹P NMR of the P(TMPY) **7**.

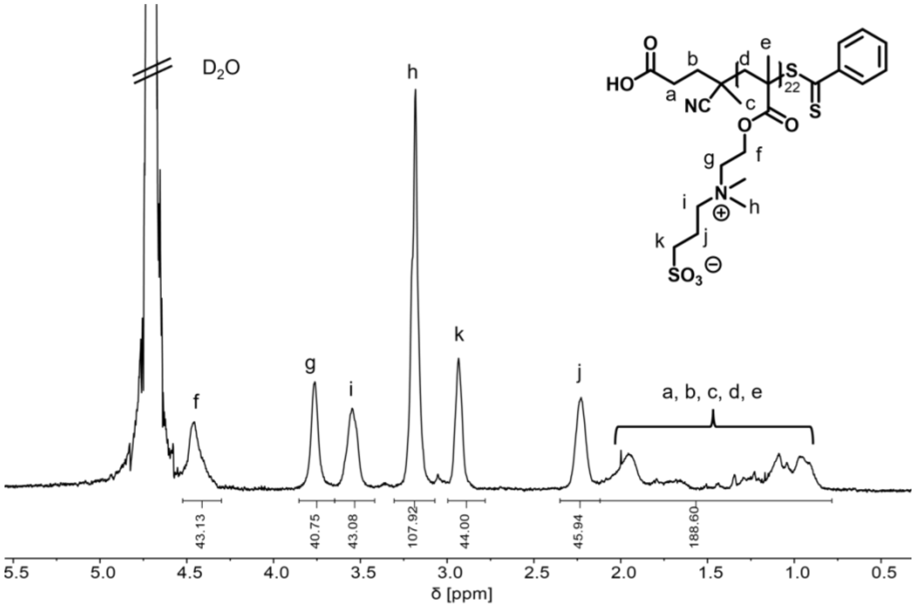

Figure S36. ^{1}H NMR spectrum of P(DMAPS) **8**.

References

(1) Frisch M. J. et al., *Gaussian 16 Rev. C.01*, Wallingford, CT, **2016**.

(2) Lee, C.; Yang, W.; Parr, R. G. Development of the Colle-Salvetti correlation-energy formula into a functional of the electron density. *Phys. Rev. B* **1988,** *37*, 785-789.

(3) Becke, A. D. Density-functional thermochemistry. III. The role of exact exchange. *J. Chem. Phys.* **1993,** *98*, 5648-5652.

(4) Grimme, S.; Antony, J.; Ehrlich, S.; Krieg, H. A consistent and accurate ab initio parametrization of density functional dispersion correction (DFT-D) for the 94 elements H-Pu. *J. Chem. Phys.* **2010,** *132*, 154104.

(5) Trobe, M.; Blesl, J.; Vareka, M.; Schreiner, T.; Breinbauer, R. A Modular Synthesis of Teraryl-Based A-Helix Mimetics, Part 4: Core Fragments with Two Halide Leaving Groups Featuring Side Chains of Proteinogenic Amino Acids. *European J. Org. Chem.* **2022**, *17*, e202101279.

(6) Drouet, F.; Noisier, A. F. M.; Harris, C. S.; Furkert, D. P.; Brimble, M. A. A Convenient Method for the Asymmetric Synthesis of Fluorinated α-Amino Acids from Alcohols. *European J. Org. Chem.* **2014**, *2014* (6), 1195–1201.

(7) Mutoh, H.; Nakamura, S.; Hagiwara, K.; Inoue, M. Construction of Pentacyclic Limonoid Skeletons via Radical Cascade Reactions. *J. Org. Chem.* **2021**, *86* (9), 6869–6878.

(8) Doncom K. E.; Willcock H.; O'Reilly R. K., *European Polymer Journal* **2017**, *87*, 497-507.

(9) Berking, B. B.; Poulladofonou, G.; Karagrigoriou, D.; Wilson, D. A.; Neumann, K. Zwitterionic Polymeric Sulfur Ylides with Minimal Charge Separation Open a New Generation of Antifouling and Bactericidal Materials, *Angew. Chem. Int. Ed.* **2023**, e202308971

(10) Van Oss, C. J.; Chaudhury, M. K.; Good, R. J. Interfacial Lifshitz-van Der Waals and Polar Interactions in Macroscopic Systems. *Chem. Rev.* **1988**, *88* (6), 927–941.

(11) Rudawska, A.; Jacniacka, E. Evaluating Uncertainty of Surface Free Energy Measurement by the van Oss-Chaudhury-Good Method. *Int. J. Adhes. Adhes.* **2018**, *82*, 139–145.

(12) Terzis, A.; Sauer, E.; Yang, G.; Groß, J.; Weigand, B. Characterisation of Acid–Base Surface Free Energy Components of Urea–Water Solutions. *Colloids Surfaces A Physicochem. Eng. Asp.* **2018**, *538*, 774–780.
